# WNT3 promotes chemoresistance to 5-Fluorouracil in oral squamous cell carcinoma via activating the canonical β-catenin pathway

**DOI:** 10.1186/s12885-024-12318-2

**Published:** 2024-05-06

**Authors:** Xuyang Zhang, Kairui Sun, Ruihuan Gan, Yuxiang Yan, Chaochao Zhang, Dali Zheng, Youguang Lu

**Affiliations:** 1 School and Hospital of Stomatology, Fujian Medical University, Fuzhou, 350004 China; 2 Fujian Key Laboratory of Oral Diseases, Fuzhou, 350004 China; 3 Fujian Provincial Biological Materials Engineering and Technology Center of Stomatology, Fuzhou, 350004 China; 4 Department of Preventive Dentistry, Hospital of Stomatology, Fujian Medical University, Fuzhou, 350002 China

**Keywords:** WNT/β-catenin pathway, Oral squamous cell carcinoma, 5-Fluorouracil, Chemoresistance, MSAB

## Abstract

**Background:**

5-Fluorouracil (5FU) is a primary chemotherapeutic agent used to treat oral squamous cell carcinoma (OSCC). However, the development of drug resistance has significantly limited its clinical application. Therefore, there is an urgent need to determine the mechanisms underlying drug resistance and identify effective targets. In recent years, the Wingless and Int-1 (WNT) signaling pathway has been increasingly studied in cancer drug resistance; however, the role of WNT3, a ligand of the canonical WNT signaling pathway, in OSCC 5FU-resistance is not clear. This study delved into this potential connection.

**Methods:**

5FU-resistant cell lines were established by gradually elevating the drug concentration in the culture medium. Differential gene expressions between parental and resistant cells underwent RNA sequencing analysis, which was then substantiated via Real-time quantitative PCR (RT-qPCR) and western blot tests. The influence of the WNT signaling on OSCC chemoresistance was ascertained through WNT3 knockdown or overexpression. The WNT inhibitor methyl 3-benzoate (MSAB) was probed for its capacity to boost 5FU efficacy.

**Results:**

In this study, the WNT/β-catenin signaling pathway was notably activated in 5FU-resistant OSCC cell lines, which was confirmed through transcriptome sequencing analysis, RT-qPCR, and western blot verification. Additionally, the key ligand responsible for pathway activation, WNT3, was identified. By knocking down WNT3 in resistant cells or overexpressing WNT3 in parental cells, we found that WNT3 promoted 5FU-resistance in OSCC. In addition, the WNT inhibitor MSAB reversed 5FU-resistance in OSCC cells.

**Conclusions:**

These data underscored the activation of the WNT/β-catenin signaling pathway in resistant cells and identified the promoting effect of WNT3 upregulation on 5FU-resistance in oral squamous carcinoma. This may provide a new therapeutic strategy for reversing 5FU-resistance in OSCC cells.

**Supplementary Information:**

The online version contains supplementary material available at 10.1186/s12885-024-12318-2.

## Background

Oral squamous cell carcinoma (OSCC) is a major malignancy within the oral and maxillofacial region [1–5]. Depending on the disease stage, treatment modalities usually include surgical resection, potentially followed by adjuvant radiotherapy or a combination of chemotherapy and radiotherapy [6, 7]. Despite implementing comprehensive strategies, the risk of recurrence following surgery and radiotherapy remains significant, as indicated by the high mortality and recurrence rates supported by histopathological evidence [8]. Adjuvant chemotherapy is indispensable for postoperative recurrence, metastasis, or advanced OSCC and is unsuitable for surgical intervention. 5-Fluorouracil (5FU), as a first-line chemotherapeutic drug, can inhibit the growth of tumor cells by inhibiting thymidylate synthase and integrating its metabolites into RNA and DNA, thus causing DNA damage [9]. Despite the remarkable efficacy of 5FU in tumor treatment, there is growing clinical evidence that the development of drug resistance severely limits its clinical potential, ultimately leading to tumor recurrence, metastasis, and subsequent treatment failure [10, 11]. A groundbreaking meta-analysis reported a modest overall survival advantage of 4% for chemotherapy at both the 2- and 5-year marks in patients with OSCC [12], highlighting that drug resistance limits improvement in overall survival [10, 13]. Therefore, it is important to determine the complex mechanisms underlying chemotherapy resistance in order to improve treatment [14].

Although there have been many studies on the mechanism of 5FU-resistance, it still remains unclear. In recent years, the Wingless and Int-1 (WNT)/β-catenin signaling pathway has received more and more attention in the study of drug resistance [15–19]. The WNT/β-catenin pathway is activated by ligands such as WNT1, WNT3, WNT3A, WNT8A, WNT10A, and WNT10B, and then β-catenin enters the nucleus and binds to the transcription factor TCF/LEF, thus regulating the expression of downstream target genes such as c-Myc, cyclin D1, and AXIN2 [20–22]. Studies have shown that abnormal activation of the WNT signaling pathway is closely related to the proliferation [23], invasion [24], metastasis [15, 16, 25], and drug resistance [26–28] of OSCC [17–19], and is a potential therapeutic target [29]. Previous studies have highlighted the effect of β-catenin on drug resistance, however, more evidence is needed to explore the effects of WNT ligands, the “switches” for pathway activation, on drug resistance. WNT3 is a ligand of the canonical WNT pathway. Many studies have investigated WNT3 in relation to other types of cancer and their chemotherapy resistance. The overall survival rate was significantly lower in non-small cell lung cancer (NSCLC) patients with high-WNT3 tumors than in those with low-WNT3 tumors [30]. Knockdown of WNT3 in human NSCLC cells suppressed cellular proliferation, invasion and metastasis, and induced apoptosis by inhibiting the canonical WNT pathway and the upregulation of WNT3 promoted cisplatin resistance [31, 32]. WNT3 contributed to cell adhesion–mediated drug resistance (CAM-DR) of myeloma cells via the WNT/RhoA/ROCK pathway of myeloma cells in an autocrine manner [33]. However, the role of WNT3 in 5FU-resistance in OSCC is unclear.

Our study found that the expression of WNT3 and its downstream target genes in the canonical WNT signaling pathway were up-regulated in 5FU-resistant OSCC cells compared to that in parental cells. To investigate the effects of these changes on drug resistance, we knocked down WNT3 in 5FU-resistant (5FU-R) cells and found that the half-maximal inhibitory concentration (IC_50_) decreased accordingly. In addition, we overexpressed WNT3 in OSCC cells and found an increase in the IC_50_, suggesting that WNT3 promoted 5FU-resistance. At the same time, it was also observed that WNT3 enhanced 5FU-resistance and promoted the proliferation and mobility of cells. In addition, we verified that upregulation of WNT3 promoted the expression of β-catenin in the nucleus, and found that the use of β-catenin inhibitor methyl 3-benzoate (MSAB) combined with 5FU reversed drug resistance. These results suggested that 5FU-resistance in OSCC might be caused by the activation of the canonical WNT signaling pathway through the upregulation of WNT3.

## Materials and methods

### Cell culture

Human OSCC cell lines (CAL27, HN30, HN6) and 293T were obtained from the School of Stomatology at Fujian Medical University. High-glucose Dulbecco’s modified Eagle medium (DMEM, Hyclone, USA) supplemented with 10% fetal bovine serum (FBS, Gibco, USA) was used for cell culture in an incubator at 37℃ in a humidified atmosphere with 5% CO_2_.

### Construction of OSCC cells with stable 5FU resistance

Parental CAL27 and HN30 cell lines were treated with increasing concentrations of 5FU (MedChemExpress (MCE), China). Over an approximate 12-month period, the 5FU-resistant cells (5FU-R) were successfully stabilized, resulting in the CAL27/5FU and HN30/5FU strains, which were then consistently nurtured in a medium supplemented with 10 µM 5FU.

### CCK8 cell viability assay

To determine the IC_50_ values of 5FU or MSAB, cells were seeded in 96-well plates in triplicate for overnight incubation and later exposed to specified drug concentrations for 48 h. To investigate the reversal of drug resistance, MSAB (MCE, China) was introduced at 0.2 µg/mL, alongside a range of 5FU concentrations. A combination chemotherapy model across a constant-ratio concentration gradient was used to determine the in vitro interaction between 5FU and MSAB at various dosages. After drug exposure, the Cell Counting Kit-8 (CCK8, Dojindo, Japan) reagent was added, and absorbance readings were captured at 450 nm on a SpectroMax iD3 microplate reader (Molecular Devices, USA).

Dose-response curves were constructed using GraphPad Prism software (version 8.0) to determine the IC_50_ values. The resistance index (RI) values were deduced by comparing the IC_50_ values of the resistant cells to those of their parental counterparts. The combination index (CI) values were determined as antagonism (CI > 1), additive effect (CI = 1), or synergism (CI < 1) by employing the Chou-Talalay method with CompuSyn software (version 1.0) [34, 35].

### Cell proliferation assay

Cells were seeded in 96-well plates and cultured for 3–5 days in the presence of the indicated drugs. Subsequently, cell viability was measured every 24 h using a CCK8 assay. Growth curves were constructed using GraphPad Prism 8.0.

### Colony formation assay

The cells were seeded in 12-well plates and incubated overnight. These cells then prospered in the presence or absence of 5FU for 2 weeks. The colonies were stained with 0.5% crystal violet solution (Beyotime, China) for photographic documentation. Colony occupation was quantified using Fiji (ImageJ) software.

### Flow cytometry analysis of cell apoptosis

Cells were seeded in 6-well plates overnight before 5FU exposure. Following treatment, cells were washed and suspended in binding buffer and stained with YF®488 Annexin V and propidium iodide (PI) (UElandy, China) for 15 min. Data acquisition was carried out on an Accuri™ C6 Flow Cytometer (BD Biosciences, USA), and subsequent analysis was executed using FlowJo software.

### Wound-healing assay

Cells were seeded into 12-well plates. Scratches were created across the monolayered cells. Wound closure in serum-free medium with 5FU was monitored and photographed (100× magnification) over the next 1–3 days, and the healing percentages were quantified using ImageJ.

### Transwell migration and invasion assay

The migration assay employed non-coated transwell® inserts with 8 μm pore size polycarbonate membranes (Corning, USA) positioned in a 24-well plate. For the invasion assay, the transwell inserts were pre-coated with Matrigel (Corning, USA) to form matrix barriers. Cell suspensions in serum-free culture medium containing 5FU were introduced into the upper chamber. The lower chamber was supplemented with 10% FBS. Following incubation at 37℃ for 48–72 h, the cells were stained using crystal violet. The lingering cells on the upper surface were removed. Stained cells were photographed (100× magnification) and quantified.

### Immunofluorescent staining and confocal microscopy

Cells grown to 50% confluence on coverslips in 12-well plates were fixed with 4% paraformaldehyde solution (Biosharp, China), followed by permeabilization using 0.5% Triton X-100 (Biofroxx, German). Ensuing steps included blocking in 3% bovine serum albumin (BSA, Biofroxx, German) and overnight incubation at 4℃ with primary antibodies against β-catenin (1:200, BD Biosciences, USA). The samples were then incubated with goat anti-mouse secondary antibody conjugated to Alexa Fluor® 488 (Cell Signaling Technology (CST), USA). In another experiment, the fixed and permeabilized cells encountered YF®594-Phalloidin (UElandy, China). The nuclei were stained with 4’,6-diamidino-2-phenylindole (DAPI, Beyotime, China). The coverslips were mounted on glass slides using a protective antifade mounting medium. Samples were observed using confocal microscope (Olympus, Japan) at 600× magnification, particularly emphasizing the nuclear translocation of β-catenin, or marking the subcellular actin.

### RNA sequencing analysis (RNA-seq) and bioinformatics analysis

Total RNA was isolated from CAL27 and CAL27/5FU cells using the TRIzol reagent (Invitrogen, USA). Library preparation and data analysis of high throughput sequencing were conducted by Novogene Co., Ltd (Beijing, China). The RNA-seq library preparations were sequenced on an Illumina Novaseq 6000 platform (Illumina, San Diego, CA). The dataset was available from the Gene Expression Omnibus (GEO) repository with accession number GSE248792. Genes with absolute fold-change > 2 in fragments per kilobase of exon model per million mapped fragments (FPKM) expression level and an adjusted P-value ≤ 0.05 found by DESeq2 were defined as differentially expressed genes (DEGs). The volcano plot and heat map presentation of DEGs and the Gene Ontology (GO) and Kyoto Encyclopedia Genes and Genomes (KEGG) pathway analyses were performed by using R language (version 4.2.1). The Cancer Genome Atlas (TCGA) database was analyzed for WNT ligand expression from TCGA- head and neck squamous cell carcinoma (HNSCC) RNAseq by using the Xiantao website (https://www.xiantaozi.com).

### Total RNA isolation and real-time quantitative PCR (RT-qPCR)

Total RNA was isolated from cells using NucleoZol (Macherey Nagel, German). Extracted RNA was reverse-transcribed into cDNAs using PrimeScript™ RT Reagent Kit with gDNA Eraser (Takara Bio, Japan), and RT-PCR was performed using the SYBR Green Kit (Bio-Rad, USA). Amplification was performed on a Real-time PCR system (Applied Biosystems, USA). The primers (Table 1) were purchased from SunYa Company (Fuzhou, China). The relative mRNA expression was normalized to the GAPDH expression using the 2^−ΔΔCt^ method.

**Table 1:** Primer sequences for target genes in RT-qPCR

Gene name (Homo sapiens)	Forward primer (5ʹ→3ʹ)	Reverse primer (5ʹ→3ʹ)
GAPDH	GGTGTGAACCATGAGAAGTATGA	GAGTCCTTCCACGATACCAAAG
WNT3	TACTCGCTCTTCAAGCCACC	CTTCTCCGTCCTCGTGTTGT
WNT5A	GCCAGTATCAATTCCGACATCG	TCACCGCGTATGTGAAGGC
WNT7A	CGCAAGCATCATCTGTAACAAG	TGAGCCTTCTCCTATGACGAT
WNT7B	GCTTTGGCGTCCTGTACGTG	AACTGGTACTGGCACTCGTTG
WNT10B	TTGTGCAGTCGGGCTCTAAG	GATGTGCAGACCCTGAAGCG
WNT11	GACCTCAAGACCCGATACCTG	TAGACGAGTTCCGAGTCCTTC
WNT16	AGTATGGCATGTGGTTCAGCA	GCGGCAGTCTACTGACATCAA
AXIN2	CCTAAAGGTCGTGTGTGGCT	ACAGTTTCCGTGGACCTCAC
CCND1	CAGACCTTCGTTGCCCTCTG	CAGTCCGGGTCACACTTGAT
CCND2	GCTGTCTCTGATCCGCAAGC	CTCAGTCAGGGCATCACAAGT
CDH1	GACCACCTTAGAGGTCAGCG	TAAGGGCTCTTTGACCACCG
CDH2	TGGGAATCCGACGAATGGATG	GAGCCACTGCCTTCATAGTCA
Vimentin (VIM)	CAGGACTCGGTGGACTTCTC	TAGTTGGCGAAGCGGTCATT

### Protein extraction and Western blot (WB)

Total protein was extracted from the samples using RIPA lysis buffer (Beyotime, China) containing protease inhibitors, PMSF and cocktail (Beyotime, China). Nuclear and cytosolic proteins were extracted using the Nuclear Protein Extraction Kit (Beyotime, China). Protein concentrations were quantified using BCA Protein Assay Kit (Beyotime, China). Equal amounts of the samples were separated by SDS-PAGE and transferred onto PVDF membranes. The membranes were blocked with 3% (w/v) BSA and incubated with primary antibodies (detailed in Table 2) overnight at 4℃. The subsequent step involved incubation with goat anti-rabbit or anti-mouse HRP-conjugated secondary antibodies (Boster, China). Visualization of protein bands was achieved with Pierce™ Enhanced Chemiluminescence (ECL) Western Blotting Substrate (Beyotime, China) on ChemiDoc™ XRS + Imaging System (Bio-Rad, USA). When the expected bands, estimated by the molecular weight ladder and the manufacturer’s instructions of primary antibodies, were separated enough, the blots were cut into 2 or 3 parts prior to incubation with primary antibodies to save the samples and primary antibodies. The original gels and multiple exposure images were shown in Supplementary file for the original images of Western blot.

**Table 2:** Information on primary antibodies used

Primary antibody	Source	Dilution
WNT3	CST	1:1000
HSP90	CST	1:1000
N-cadherin (N-Cad)	CST	1:1000
Vimentin (VIM)	CST	1:1000
GAPDH	CST	1:1000
β-catenin	CST	1:1000
LaminA/C	CST	1:1000
Flag	CST	1:1000
Caspase-3	CST	1:1000
c-Caspase-3	CST	1:1000
PARP	CST	1:1000
c-PARP	CST	1:1000
α-tubulin	CST	1:1000

### Small interfering RNA (siRNA) transfection

The sequences of siRNAs (GenePharma, China) targeting WNT3 (siWNT3) and the negative control (siNC) are detailed in Table 3. Resistant cells were seeded in 6-well plates and transiently transfected with siRNAs using the Lipofectamine RNAiMAX Transfection Reagent (Invitrogen, USA). 48 h and 72 h after transfection, both RNA and proteins were harvested for subsequent assays. Cells for subsequent functional assays were processed on the day after transfection.

**Table 3:** siRNA sequences targeting WNT3

Sequences	Sense (5ʹ→3ʹ)	Antisense (5ʹ→3ʹ)
siWNT3-646	GCCAUGAACAAGCACAACATT	UGUUGUGCUUGUUCAUGGCTT
siWNT3-1120	CAGGAGUGUAUUCGCAUCUTT	AGAUGCGAAUACACUCCUGTT
siNC	UUCUCCGAACGUGUCACGUTT	ACGUGACACGUUCGGAGAATT

### Transient plasmid transfection

The overexpression plasmid WNT3-Flag (WNT3) and its corresponding control (vector) were obtained from Dahong Biotechnology (Guangzhou, China). After seeding OSCC cells in 6-well plates, they were transiently transfected with plasmids using the Lipofectamine 2000 reagent (Invitrogen, USA) and the medium was replaced with fresh medium. Post-transfection protein analysis verified the overexpression efficiency, and the cells were subsequently used for additional experiments.

### Dual-luciferase reporter assay

293T cells at 80% confluence were co-transfected with the WNT3 plasmid or its control vector with Top-flash (Addgene, USA) and Renilla plasmids (Promega, USA) using the Lipofectamine 2000. After 48 h, cell lysates were harvested. Luciferase activity was tested using the Dual Luciferase Assay System (Yeason, China), with firefly luciferase activity normalized to that of Renilla luciferase.

### Statistical analysis

All data are presented as mean ± standard deviation, as determined using GraphPad Prism 8.0. Statistical analyses were performed using Student’s t-test, with *P* < 0.05 as statistically significant (ns = not significant, ^*^ *P* < 0.05, ^**^ *P* < 0.01, ^***^ *P* < 0.001).

## Results

### Establishment and characterization of 5FU‑resistant OSCC cells

**Fig. 1:**
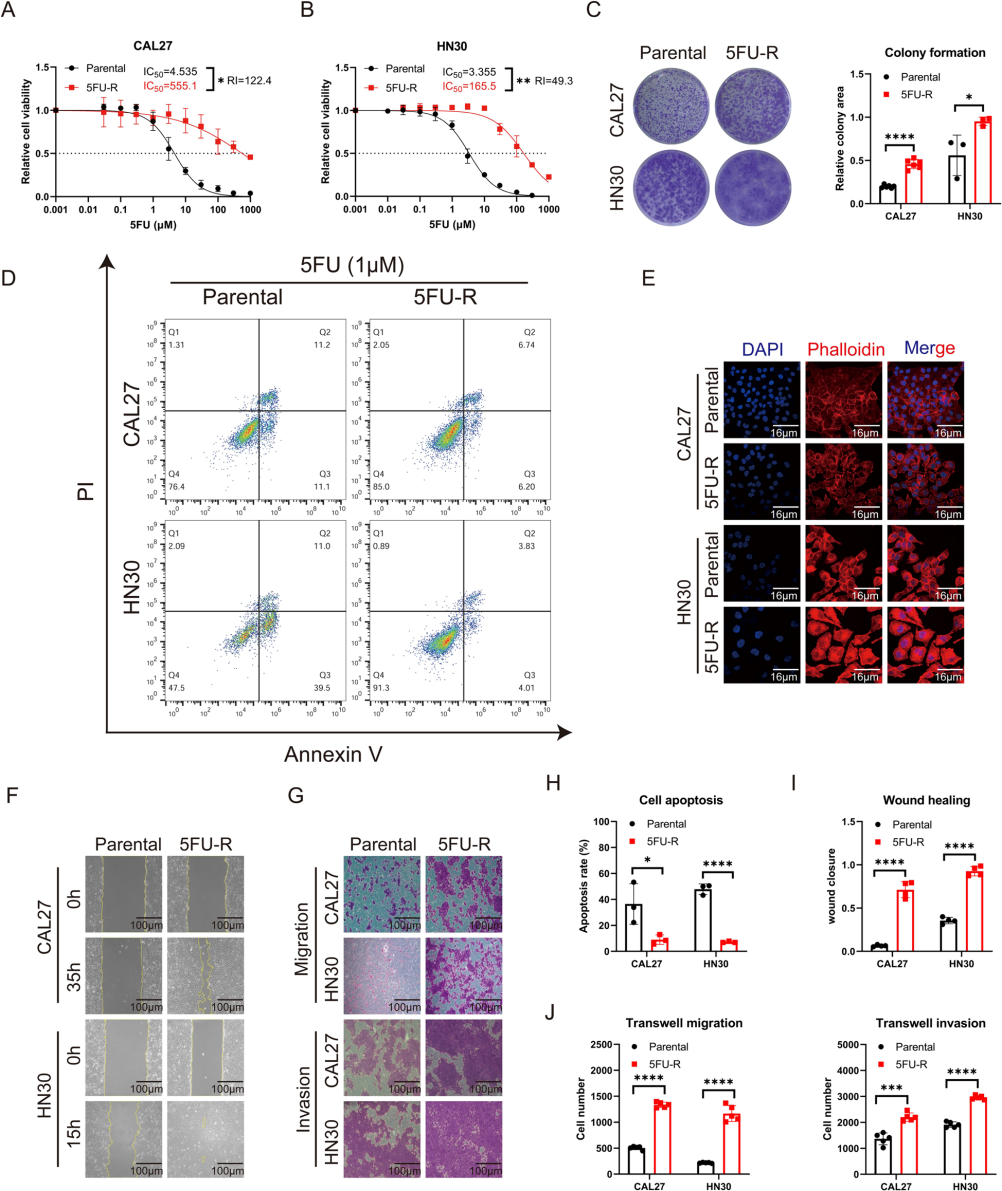
Establishment and characterization of 5FU-resistant (5FU-R) oral squamous cell carcinoma (OSCC) cells. **A**, **B** Cell viabilities and IC_50_ of 5FU comparing parental and 5FU-R cells via CCK8 assay. **C** Colony-forming potential of parental and 5FU-R cells. **D** and **H** Apoptosis under 5FU exposure in parental and 5FU-R cells. **E** Cytoskeletal configurations (600×) in parental and 5FU-R cells visualized with F-actin staining by YF®594-Phalloidin (red) and nuclear staining by DAPI (blue). **F** and **I** Wound-healing (100×) in parental and 5FU-R cells. **G** and **J** Migration and invasion (100×) of parental and 5FU-R cells

Therapeutic resistance is of paramount importance in OSCC treatment. To elucidate the 5FU-resistance mechanism, we established 5FU-resistant cells. We first assessed the viability of the parental and resistant cells after 5FU exposure. The CCK8 assays emphasized a dose-dependent reduction in OSCC cell viability after 5FU. Among them, the IC_50_ values of 5FU in parental cells and 5FU-R cells were 4.535 µM and 555.1 µM, respectively, for the CAL27 cell line, and 3.355 µM and 165.5 µM for the HN30 cell line, respectively (Fig. 1A, B). The 5FU-R cells displayed a RI of 122.4 for CAL27 and 49.3 for HN30 cell line. Additionally, the clonogenic potential of 5FU-R cells surpassed that of the parental cells (Fig. 1C). When subjected to 5FU, the apoptosis rate at 1 µM 5FU was notably higher in parental cells compared to 5FU-R cells (Fig. 1D, H). Phalloidin staining showed that 5FU-R cells were more dispersed, with increased intercellular gaps and fewer connections compared to the parental cells (Fig. 1E). On assessing other phenotypes via wound-healing, transwell migration and invasion assays, we found enhanced migration and invasion capacities in 5FU-R cells compared to parental cells (Fig. 1F, G, I, J).

We successfully established an in vitro resistant cell model and demonstrated that 5FU-R OSCC cells possessed heightened resistance, proliferation, and metastatic capabilities.

### Identification of WNT3 as a contributor of 5FU-resistance by activating the β-catenin signaling pathway in OSCC cells

**Fig. 2:**
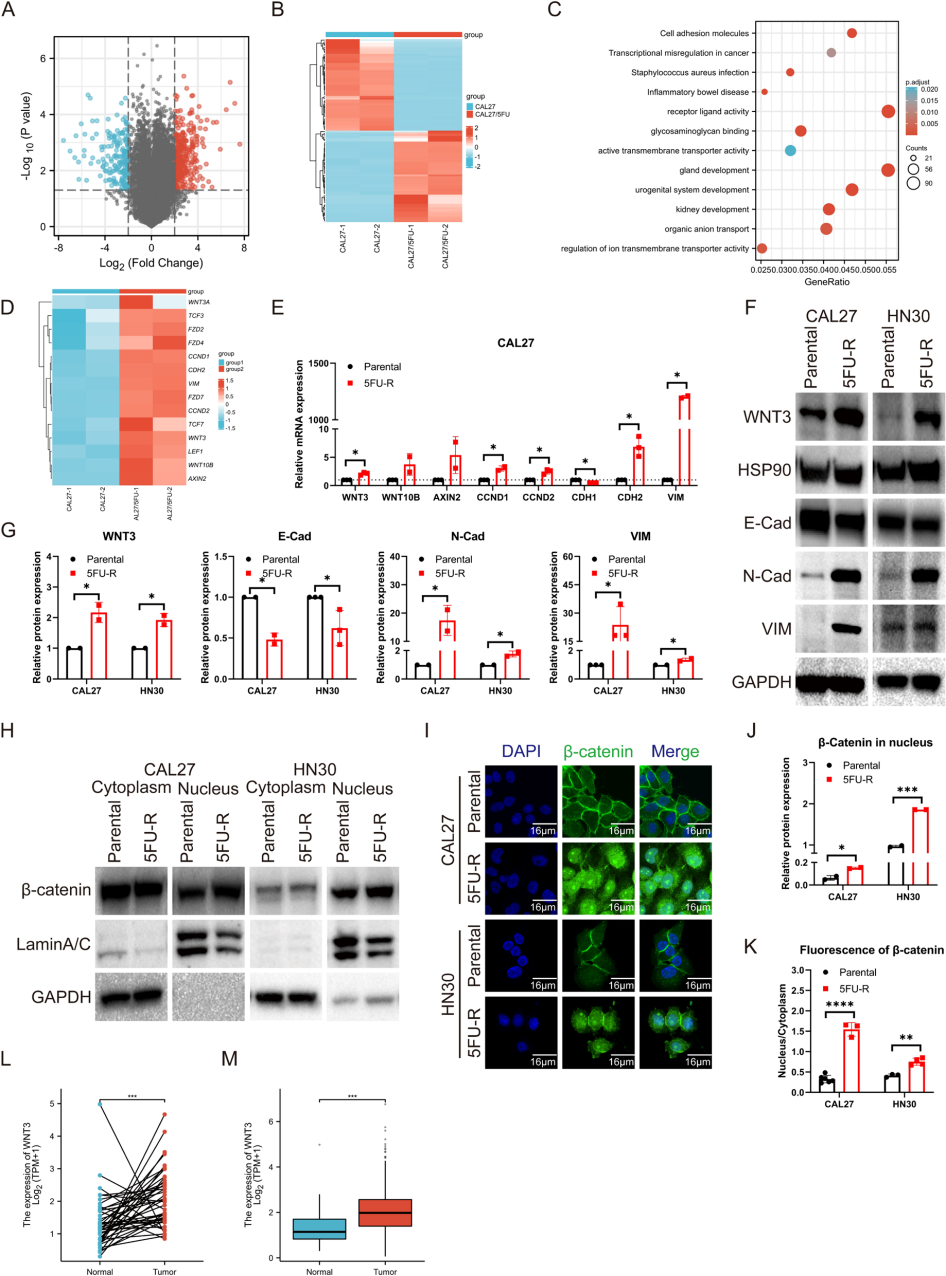
Transcriptomic analysis and in vitro validation for RNA-seq. **A** Volcano plot illustrating gene expression differences between parental and 5FU-R cells. **B** Segment of hierarchical clustering for parental and 5FU-R cells. **C** GO and KEGG enrichment analyses for DEGs between parental and 5FU-R cells. **D** Heat map representation of genes related to the WNT pathway. **E** RT-qPCR validation for changes in the expression of WNT pathway-associated genes. **F**, **G** Western blot analyses for WNT3, E-Cad, N-cad, and VIM protein levels. **H** and **J** Differential expression of β-catenin in the cytoplasm and nucleus of parental and 5FU-R cells by western blot. **I** and **K** Immunocytochemical imaging (600×) of β-catenin (green) with nuclear staining by DAPI (blue). **L**-**M** WNT3 expression in HNSCC based on the TCGA database

To uncover the pivotal factors for 5FU-resistance in oral cancer, we extracted RNA and employed RNA-seq to identify key differentially expressed genes (DEGs) between the parental and 5FU-resistant cells. The volcano plot illustrated the decreased expression of 598 genes (blue dots) and increased expression of 1221 genes (red dots), with gray dots indicating negligible variation (Fig. 2A). A heatmap was generated to assess the overall gene expression across the samples (Fig. 2B). Further exploration of DEGs associated with tumor-related biological processes and signaling pathways was performed using Gene Ontology (GO) and Kyoto Encyclopedia Genes and Genomes (KEGG) enrichment analyses, focusing on the CAL27 and CAL27/5FU samples (Fig. 2C).

Interestingly, transcriptome sequencing highlighted abnormal activation of the WNT signaling pathway in 5FU-resistant OSCC cells. A heatmap of genes associated with the WNT signaling pathway revealed marked upregulation of several WNT ligands, transcription factors, and downstream effectors. Conversely, genes linked to the degradation complex were downregulated in CAL27/5FU cells compared with those in CAL27 cells (Fig. 2D). To validate these findings, we selected candidate genes for RT-qPCR analysis, which revealed a surge in the expression of WNT3, CCND1, CCND2, CDH2, and Vimentin (VIM) in 5FU-R cells, while CDH1 expression declined (Fig. 2E). No significant variations were observed in the remaining genes in either cell type. Moreover, the protein levels of WNT3, E-cadherin (E-Cad), N-cadherin (N-Cad), and VIM were upregulated in 5FU-R cells (Fig. 2F, G). Therefore, we speculated that the WNT3 and WNT pathway might be associated with resistance to 5FU in oral cancer.

Conventionally, the canonical WNT signaling pathway gets triggered and stabilized by β-catenin. In this scenario, non-phosphorylated β-catenin permeates the nuclei, activating the downstream target genes of the WNT/β-catenin pathway. To identify the specific WNT signaling pathway involved, we evaluated the nuclear β-catenin protein levels by western blot and verified its nuclear translocation by immunofluorescence. Elevated nuclear β-catenin expression was detected in 5FU-R cells (Fig. 2H, J). Immunofluorescence further substantiated this, showing augmented nuclear localization in 5FU-R cells due to the translocation of activated β-catenin, which predominantly resided in the cytoplasm in parental cells (Fig. 2I, K). The data from TCGA showed a predominant elevation in HNSCC genes (Fig. 2L, M, partial data). Notably, WNT3 is a ligand of WNT/β-catenin as a switch in the WNT canonical path [36] and has been generally linked to OSCC progression in previous studies [37], but no association with drug resistance has been reported. In our study, 5FU-R cells showed elevated mRNA and protein levels of WNT3 (Fig. 2E-G). Given its role as an upstream ligand of β-catenin in the WNT pathway, we hypothesized that the activation of the WNT/β-catenin pathway observed in 5FU-R cells could be attributed to amplified WNT3 expression. Consequently, under the stress of 5FU chemotherapy, OSCC cells may bolster their survival by enhancing WNT3 expression.

Therefore, in the following experiments, we manipulated WNT3 using siRNAs and WNT3 plasmids to gain further insight into the molecular complexity of how WNT3 regulated 5FU-resistance.

### Downregulation of WNT3 enhances 5FU efficacy in 5FU-resistant OSCC cells

**Fig. 3:**
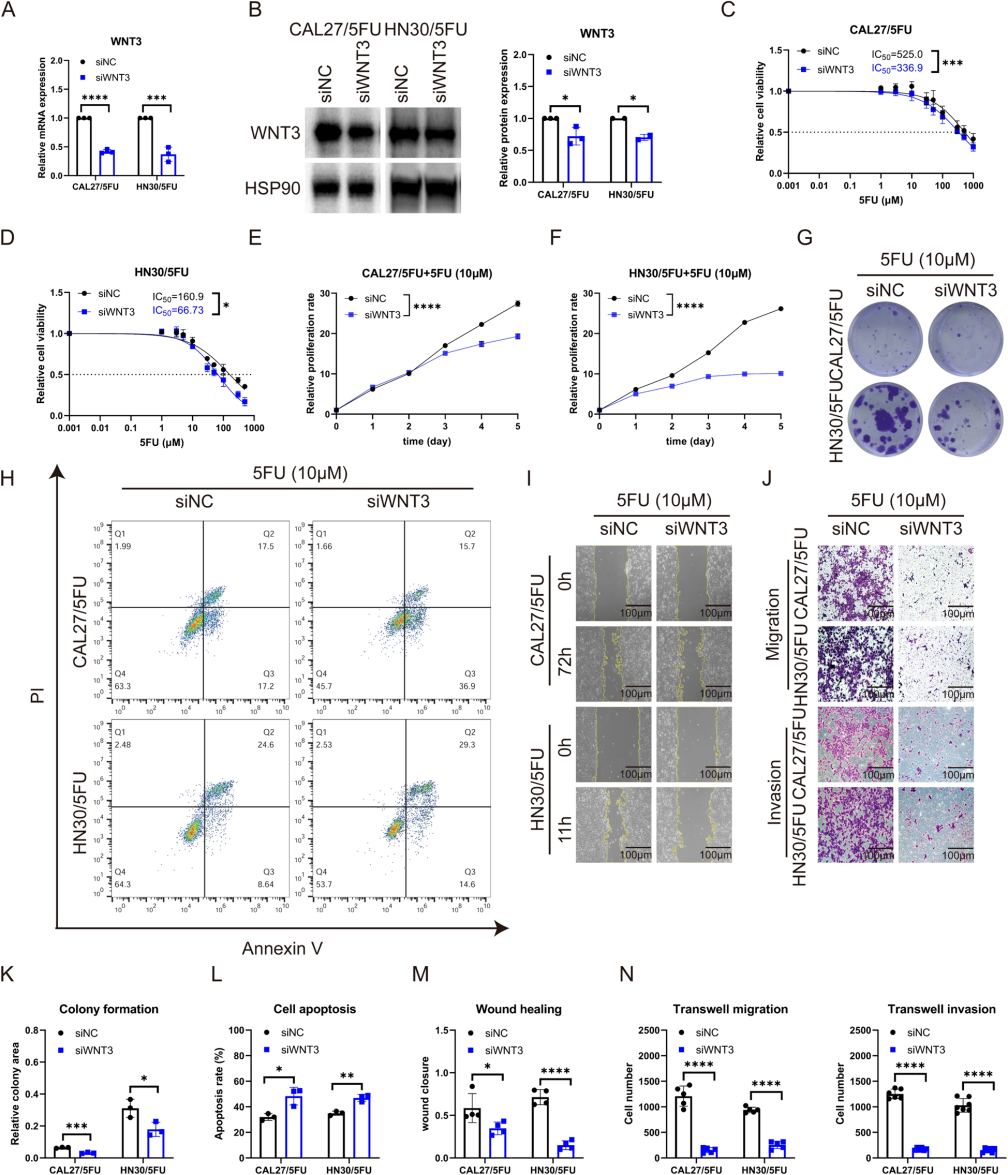
Downregulation of WNT3 enhances 5FU efficacy in 5FU-resistant OSCC cells. **A**, **B** RT-qPCR and western blot evaluations of WNT3 expression post-knockdown in 5FU-R cells. **C**, **D** Decreased IC_50_ of 5FU in 5FU-R cells after WNT3 knockdown. **E**, **F** Inhibited proliferation under 5FU exposure in 5FU-R cells after transfection with siWNT3. **G** and **K** Reduced colony-forming potential under 5FU exposure in 5FU-R cells after transfection with siWNT3. **H** and **L** Increased apoptosis under 5FU exposure in 5FU-R cells following WNT3 knockdown. **I** and **M** Decreased wound-healing (100×) under 5FU exposure in 5FU-R cells after WNT3 knockdown. **J** and **N** Diminished migration and invasion (100×) under 5FU exposure in 5FU-R cells after WNT3 knockdown

 To ascertain the role of WNT3 in resistance to 5FU in OSCC cells, we suppressed the endogenous expression of WNT3 in 5FU-resistant CAL27 and HN30 cells using siRNA constructs. The knock-down effect of the two siRNA sequences (siWNT3-646 and siWNT3-1120) targeting WNT3 verified by RT-qPCR was very good in 5FU-R cells (Fig S1). According to Dharmacon™ siRNA solutions (https://horizondiscovery.com/en/gene-modulation/knockdown/sirna), pooling siRNA improves effectiveness. Therefore, in subsequent experiments, the two sequences were equally mixed as transfection mixture of siWNT3 group, and the knockdown efficiency at the mRNA and protein levels in 5FU-R cells was validated (Fig. 3A, B). Subsequent 5FU treatment of the control and WNT3-silenced cells revealed a partial reversal of 5FU-resistance with WNT3 knockdown. Notably, the IC_50_ values decreased from 525.0 µM to 336.9 µM for CAL27/5FU and from 160.9 µM to 66.73 µM for HN30/5FU (Fig. 3C, D). Furthermore, WNT3 knockdown enhanced the inhibitory effect of 5FU on the proliferation of resistant OSCC cells (Fig. 3E-F) and reduced the colony-forming potential of 5FU-R cells (Fig. 3G, K). After WNT3 silencing, the apoptosis rate of drug-resistant cells increased in the presence of 5FU (Fig. 3H, L). We analyzed whether changes in WNT3 affected the inhibitory effect of 5FU on the mobility of drug-resistant cells. We found that downregulation of WNT3 hindered cell migration under the action of 5FU through wound healing and transwell migration assays and inhibited cell invasion, as confirmed by the transwell invasion assay (Fig. 3I, J, M, N).

These findings suggested that WNT3 played a pivotal role in 5FU-resistance in OSCC cells. Its suppression restored drug sensitivity and inhibited growth, migration, and invasion of these cells.

### Upregulation of WNT3 augments 5FU-resistance in OSCC cells

**Fig. 4:**
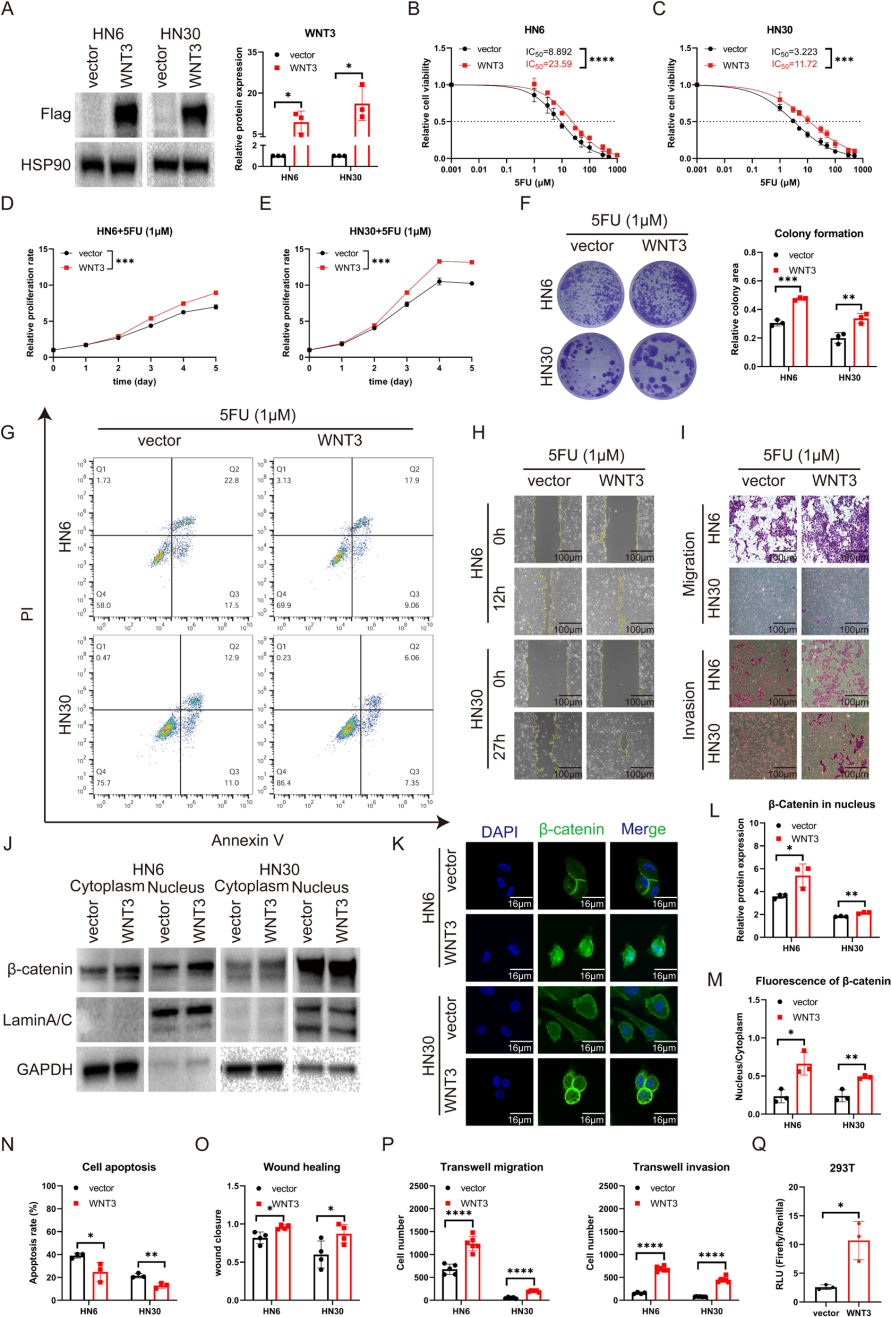
Upregulation of WNT3 augments 5FU-resistance in OSCC cells. **A** Western blot determinations of WNT3 protein levels in OSCC cells after overexpression of WNT3. **B**, **C** Elevated IC_50_ of 5FU in OSCC cells post-WNT3 upregulation. **D**, **E** Enhanced proliferation under 5FU exposure in OSCC cells transfected with WNT3. **F** Enhanced colony-forming ability under 5FU exposure in OSCC cells after WNT3-overexpression. **G** and **N** Reduced apoptosis under 5FU exposure in OSCC cells with WNT3 overexpression. **H** and **O** Accelerated wound-healing (100×) under 5FU exposure in OSCC cells after WNT3-overexpression. **I** and **P** Elevated migration and invasion (100×) under 5FU exposure in OSCC cells after WNT3 upregulation. **J** and **L** Differential expression of β-catenin in the cytoplasm and nucleus of OSCC cells after overexpression of WNT3 by western blot. **K** and **M** Immunocytochemical imaging (600×) of β-catenin (green) with nuclear counterstaining by DAPI (blue). **Q** Enhanced luciferase activity in dual-luciferase reporter assay after WNT3 overexpression

 To verify the hypothesis that WNT3 supports chemoresistance to 5FU, we overexpressed WNT3 in parental OSCC cells by transfection with WNT3 plasmids. First of all, the expression levels of endogenous WNT3 in CAL27, HN6, and HN30 cells were detected by western blot. We found that the expression of WNT3 in HN6 and HN30 cells was lower than that in CAL27 cells (Fig S2). Considering the poor transfection results of overexpression in parental CAL27 cells in the previous preliminary experiment, overexpression in cells with low expression level would achieve better results and HN6 and HN30 cells were used in this study. Western blot analysis confirmed a notable increase in WNT3 protein levels after transfection (Fig. 4A).

After upregulation of WNT3, we detected a rise in 5FU-resistance: the IC_50_ value shifted from 8.892 µM to 23.59 µM in HN6 and from 3.223 µM to 11.72 µM in HN30 (Fig. 4B, C). Additionally, WNT3 overexpression promoted cell proliferation and colony formation in response to 5FU (Fig. 4D-F), indicating that high expression of WNT3 protected OSCC cells from the anti-proliferative effects of 5FU. Consistent with these observations, WNT3 overexpression conferred resistance to 5FU-induced apoptosis (Fig. 4G, N). Further investigation revealed that WNT3 also accelerated cell mobility under the action of 5FU. Wound healing and transwell assays showed that the upregulation of WNT3 expression promoted wound closure and cell invasion and migration in the presence of 5FU observed in HN6 and HN30 cells (Fig. 4H, I, O, P).

Does the effect of WNT3 on drug resistance in OSCC arise through the activation of the canonical WNT signaling pathway? We verified the activation of the canonical signaling pathway by WNT3 using nucleoplasmic isolation, immunofluorescence, and dual-luciferase reporter assays. The results showed that nuclear β-catenin levels also surged (Fig. 4J, L). Using immunofluorescence techniques, we observed the transfer of β-catenin from the cytoplasm to the nucleus following WNT3 overexpression (Fig. 4K, M), which marked the key transcriptional activation step. This was further confirmed by the enhanced luciferase activity in the dual-luciferase reporter assay after the induction of WNT3 (Fig. 4Q).

Our data emphasized that WNT3 enhanced the resistance to 5FU in OSCC through the β-catenin signaling pathway, promoted cell proliferation and mobility under 5FU action, and reduced the level of apoptosis.

### WNT pathway inhibitor MSAB increases 5FU efficacy in 5FU-resistant OSCC cells

We have previously demonstrated that increased WNT3 expression amplified 5FU-resistance in OSCC cells and activated the canonical WNT signaling pathway. To ascertain whether the β-catenin pathway could alter the chemotherapy-resistant phenotype of 5FU-R cells, we treated 5FU-R cells with WNT/β-catenin signaling inhibitor MSAB [38] to investigate whether combining 5FU with a WNT-specific inhibitor would improve its efficacy.

**Fig. 5:**
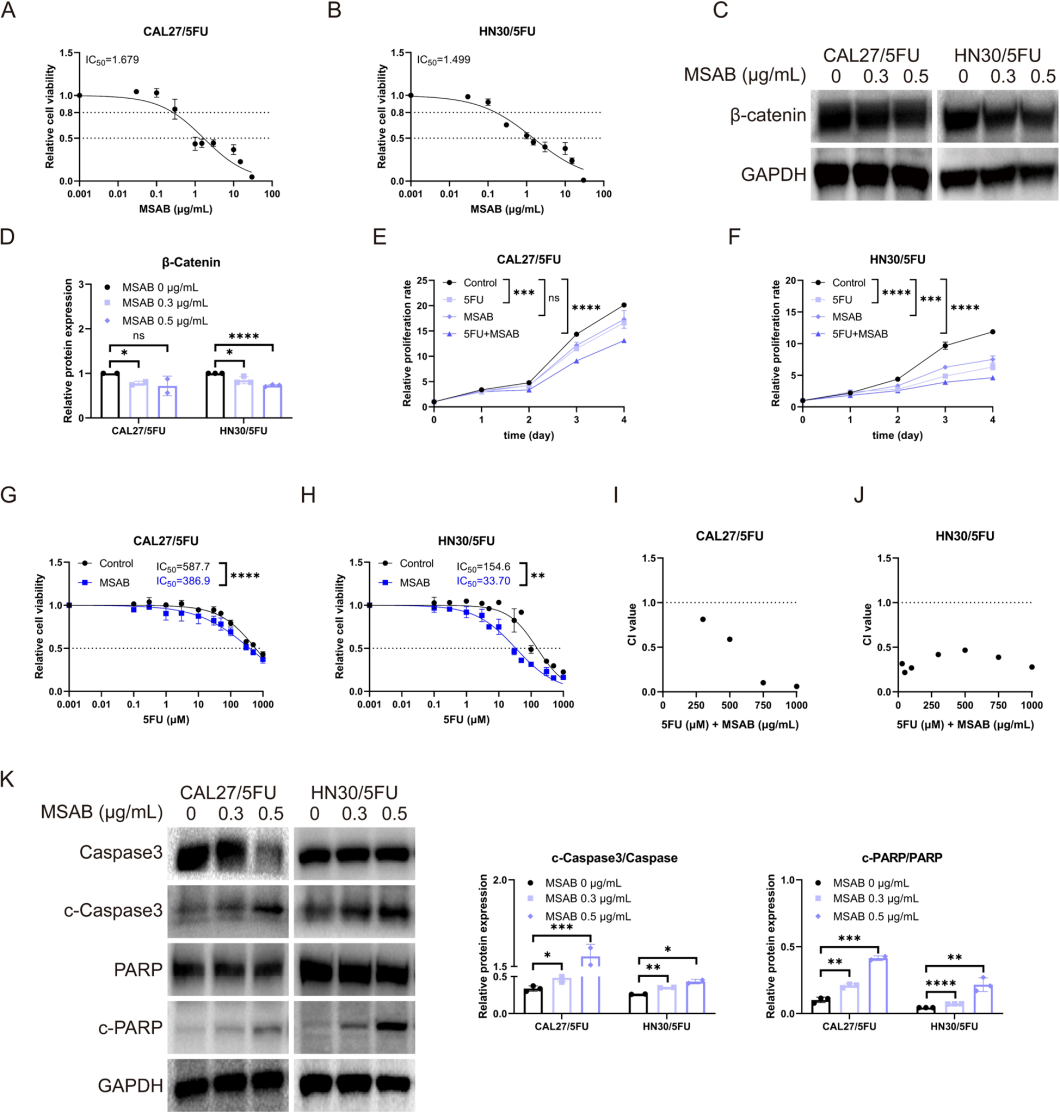
MSAB increases 5FU efficacy in 5FU-resistant OSCC cells. **A**, **B** Cell viabilities and IC_50_ of MSAB on 5FU-R cells. **C**, **D** Decreased β-catenin protein levels after MSAB treatment. **E**, **F** Inhibited proliferation of 5FU-R cells when treated with MSAB combined with 5FU. **G**, **H** Decreased IC_50_ of 5FU in 5FU-R cells when combined with 0.2 µg/mL MSAB. **I**, **J** Synergistic effects as indicated by CI < 1 when combining MSAB and 5FU. **K** Upregulation of apoptosis-related proteins after MSAB treatment

The optimal MSAB concentration for this experiment was determined by the CCK8 test. MSAB reduced the viability of 5FU-R cells in a dose-dependent manner (Fig. 5A, B). Concurrently, the administration of MSAB resulted in a decrease in β-catenin protein levels (Fig. 5C-D). In subsequent experiments combining chemotherapy with MSAB, we observed a significant inhibition of 5FU-R cell proliferation (Fig. 5E, F). Specifically, treatment in combination with MSAB resulted in a more pronounced reduction in cell viability compared to the control cells and those receiving monotherapy. This suggested that the introduction of MSAB enhanced the growth inhibitory effect of 5FU. Co-treatment with MSAB (MSAB group) diminished 5FU-resistance in both CAL27/5FU and HN30/5FU cell lines compared with the control group, with the IC_50_ of 5FU dropping from 587.7 µM to 386.9 µM and from 154.6 µM to 33.70 µM, respectively (Fig. 5G, H). This underscored the notion that β-catenin suppression reduced 5FU-resistance. In a further study involving co-treatment with MSAB and 5FU, we evaluated the CI values of 5FU alongside MSAB. CI values below 1 indicated a synergistic effect of MSAB and 5FU (Fig. 5I, J). In addition, MSAB treatment increased the expression of cleaved Caspase-3, subsequently accelerating the hydrolysis of its substrate poly (ADP-ribose) polymerase (PARP) (Fig. 5K), suggesting that MSAB induced apoptosis in 5FU-R cells.

Our results suggested that disrupting the WNT pathway enhanced the sensitivity of 5FU-R cells to 5FU. The therapeutic combination of a WNT inhibitor and 5FU enhanced the treatment potency of 5FU, further emphasizing the pivotal role of β-catenin inhibition in counteracting 5FU-resistance.

## Discussion

Chemotherapy serves as a supplementary treatment for most patients with oral cancer following surgical procedures. As a first-line therapeutic agent in both chemotherapy alone and combination chemotherapy regimens, 5FU is integral to oral cancer treatment [39]. However, the emergence of 5FU-resistance in many patients underscores the urgent need for effective countermeasures. Despite numerous molecular-targeted strategies proposed to address 5FU-resistance in OSCC, practical solutions remain elusive. A growing body of evidence highlights the activation or hyperactivation of WNT/β-catenin in the oncogenesis of various human cancers [40–43]. Previous studies have identified frequent upregulation and mutations in various WNT family members in diverse cancer types [44]. In addition, an association between the WNT signaling pathway and OSCC prognosis has been established [45–52], positioning it as an independent prognostic factor [16, 53, 54]. The role of WNT/β-catenin activation in mediating chemoresistance in other cancers has also been confirmed in the emerging literature [18, 55, 56], in which WNT/β-catenin activation supports 5FU-resistance through mechanisms such as cancer stem cell, metastasis, and enhanced transcriptional activity [19, 57–59].

WNT3, a ligand in the WNT signaling pathway that drives canonical WNT/β-catenin signaling through autocrine or paracrine mechanisms, plays an important role in various biological activities, and has been studied in the context of drug resistance in other cancers. For example, WNT3 silencing inhibits cell proliferation and enhances cell sensitivity to Cisplatin in NSCLC [32]. Furthermore, activation of WNT3 was observed to stimulate the WNT/β-catenin pathway and an epithelial-mesenchymal transition (EMT)-like phenotype in trastuzumab-resistant HER2-overexpressing breast cancer cells [60]. However, the effect of WNT3 on 5FU-resistance in OSCC and related mechanisms have not been reported. Our results confirm the role of WNT3 in promoting the progression of 5FU-resistant OSCC cells, providing more evidence for the role of WNT signaling pathway in chemoresistance in OSCC. In the transcriptome sequencing of 5FU-resistant cells constructed from OSCC, we found an upregulation of WNT3 expression and aberrant activation of the WNT pathway, which drew our attention to the question of whether 5FU-resistance in OSCC is related to these abnormal manifestations. We verified our hypothesis through a seriels of experiments. It is important to note that while WNT3 is a canonical ligand of the WNT pathway, it does not necessarily imply that the activation of the WNT pathway is solely attributable to WNT3. We therefore further determined the WNT3-induced activation of the canonical signal pathway in OSCC cells through the increased levels of intranuclear β-catenin protein and the translocation of β-catenin into the nucleus in immunofluorescence and luciferase reporter gene assays.

It is also possible to question whether other signaling pathways are involved in 5FU-resistance in OSCC. In fact, in the initial analysis of the sequencing results, we observed the most pronounced changes in the WNT pathway, which was selected as the research object for this study. Other signaling pathways may be involved; however, it is critical to recognize the complexity of replicating tumor cell environments in vivo and in vitro. Although this study is insightful, it has some inherent limitations. A more nuanced understanding of drug resistance mechanisms requires extensive experimentation. At present, our team is making related efforts such as constructing drug-resistant patient-derived xenograft (PDX) mouse models by mimicking the human drug-resistance process and exploring related drug-resistance mechanisms.

Notably, N-cadherin (N-Cad, encoded by the CDH2 gene), an EMT marker recognized as being critical for the diagnosis and prognosis of breast cancer [61], was upregulated in this study. Many studies have shown that cells undergoing EMT frequently exhibit amplified EMT traits during drug resistance [62], which was also reflected in our experimental results. In the PDX-resistant mouse model being constructed by our research group, proteins in the tumor tissues were extracted during the process of gradual induction, and EMT changes in the 5FU treatment group were also detected (relevant results not shown), suggesting that the mobility of tumor cells was stimulated during the development of acquired drug resistance. However, the mechanisms underlying these changes require further investigation. Current therapeutic strategies targeting WNT signaling include small-molecule inhibitors targeting β-catenin/TCF signaling activity and antibodies against WNT1 and WNT2 [63]. In vitro experiments and in mouse tumor models, MSAB, the inhibitor of WNT/β-catenin signaling pathway, showed significant selective antitumor effects by inhibiting target genes in colorectal cancer cells [38]. Our observations showed that MSAB-treated 5FU-R cells regained sensitivity to 5FU. MSAB intervention attenuated cell proliferation and increased the rate of apoptosis, as evidenced by the ensuing cascade of caspase activation. These findings underscored the critical role of the WNT/β-catenin signaling pathway in the dynamics of resistance to 5FU in OSCC. Recent studies advocate the specific targeting of WNT3, indicating its potential in countering Trastuzumab resistance, which may be of therapeutic benefit for patients with breast cancer overexpressing HER2 [60]. Therefore, the development of treatments targeting WNT3 is a promising frontier in oncology research.

## Conclusions

In conclusion, by constructing 5FU-resistant OSCC cell lines, we revealed the upregulation of WNT3 expression in resistant cells, activating the WNT canonical signaling pathway, and we validated the promoting effect of WNT3 on 5FU resistance. At the same time, we further verified the reversal effect of 5FU combined with MSAB on resistance in OSCC. Our work has demonstrated the role of WNT3 in promoting 5FU resistance in OSCC, therefore, targeting WNT3 therapy may provide a promising strategy for improving 5FU sensitivity in the treatment of OSCC.

### Supplementary Information

Supplementary Material 1.

Supplementary Material 2.

## Data Availability

The RNA-seq dataset was uploaded to the GEO online repository with the number GSE248792. All the data presented in this study and information supporting the results can be found in the article or in the supplementary information files and are available upon reasonable request from the corresponding authors.

## Abbreviations

5FU: 5-Fluorouracil
5FU-R: 5FU-resistant cells
BSA: Bovine serum albumin
CAM-DR: Cell adhesion–mediated drug resistance
CCK8: The Cell Counting Kit-8
CI: The combination index values
DAPI: 4',6-diamidino-2-phenylindole
DEGs: Differentially expressed genes
DMEM: Dulbecco's modified Eagle medium\
ECL: Enhanced Chemiluminescence
EMT: Epithelial-mesenchymal transition
FBS: Fetal bovine serum
FPKM: Fragments per kilobase of exon model per million mapped fragments
GEO: The Gene Expression Omnibus repository
GO: Gene Ontology
HNSCC: Head and neck squamous cell carcinoma
IC_50_: The half-maximal inhibitory concentration
KEGG: Kyoto Encyclopedia Genes and Genomes
MSAB: Methyl 3-benzoate
NC: Negative control
N-Cad: N-cadherin
NSCLC: Non-small cell lung cancer
OSCC: Oral squamous cell carcinoma
PARP: Poly (ADP-ribose) polymerase
PDX: Patient-derived xenograft
PI: Propidium iodide
RI: The resistance index values
RNA-seq: RNA sequencing
RT-qPCR: Real-time quantitative PCR
siRNA: Small interfering RNA
TCGA: The Cancer Genome Atlas database
VIM: Vimentin
WB: Western blot
WNT: The Wingless and Int-1
