# Supplementary material for: WNT3 promotes chemoresistance to 5-Fluorouracil in oral squamous cell carcinoma via activating the canonical β-catenin pathway
Source: BMC Cancer. 2024 May 6;24:564. doi: 10.1186/s12885-024-12318-2 (PMC11071218; doi:10.1186/s12885-024-12318-2)
Supplement: Supplementary file 1 — Supplementary Material 1. [file 12885_2024_12318_MOESM1_ESM.pdf]

Supplementary figures

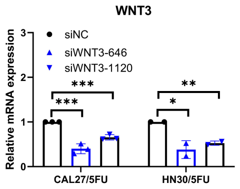

Fig. S 1 The knock-down efficiency of two siRNA sequences targeting WNT3 verified by qPCR in 5FU-R cells

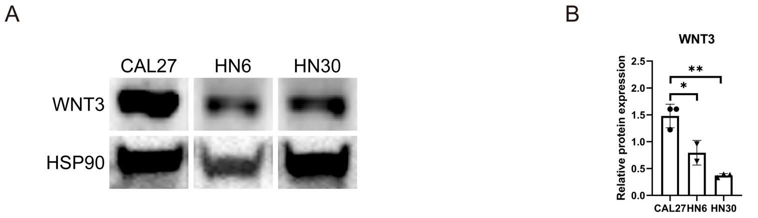

Fig. S 2 Western blot analysis (A-B) of endogenous WNT3 protein levels in OSCC cells

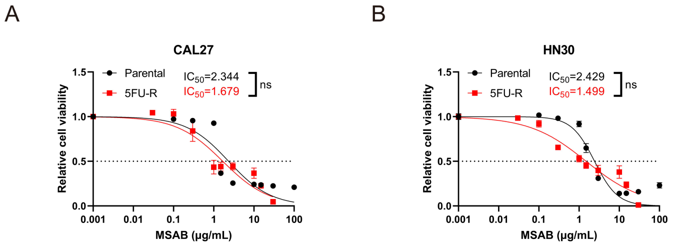

Fig. S 3 Cell viabilities and IC_{50} of MSAB on Parental and 5FU-R cells in CAL27 (A) and HN30 (B) cell lines
