# Supplementary figures and images for: WNT3 promotes chemoresistance to 5-Fluorouracil in oral squamous cell carcinoma via activating the canonical β-catenin pathway
Source: BMC Cancer. 2024 May 6;24:564. doi: 10.1186/s12885-024-12318-2 (PMC11071218; doi:10.1186/s12885-024-12318-2)

Figure 2F

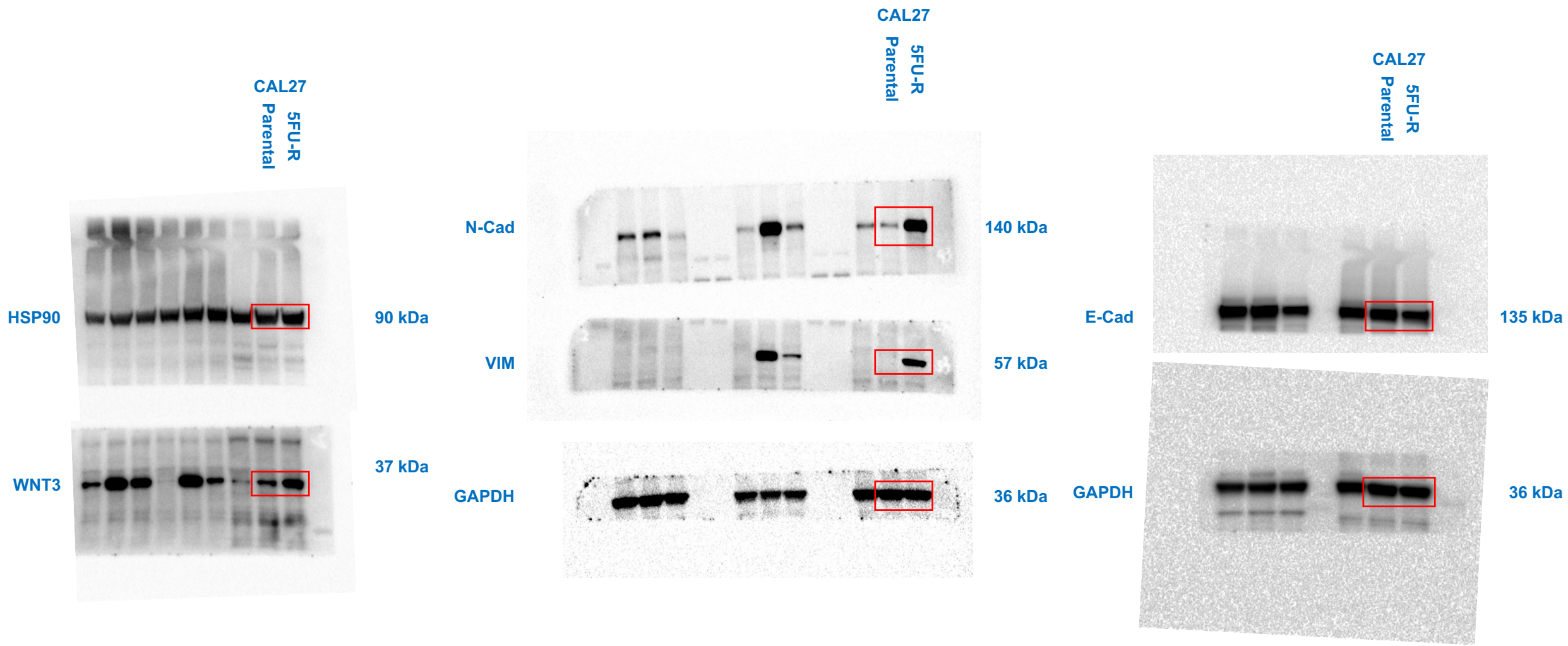

Figure 2F

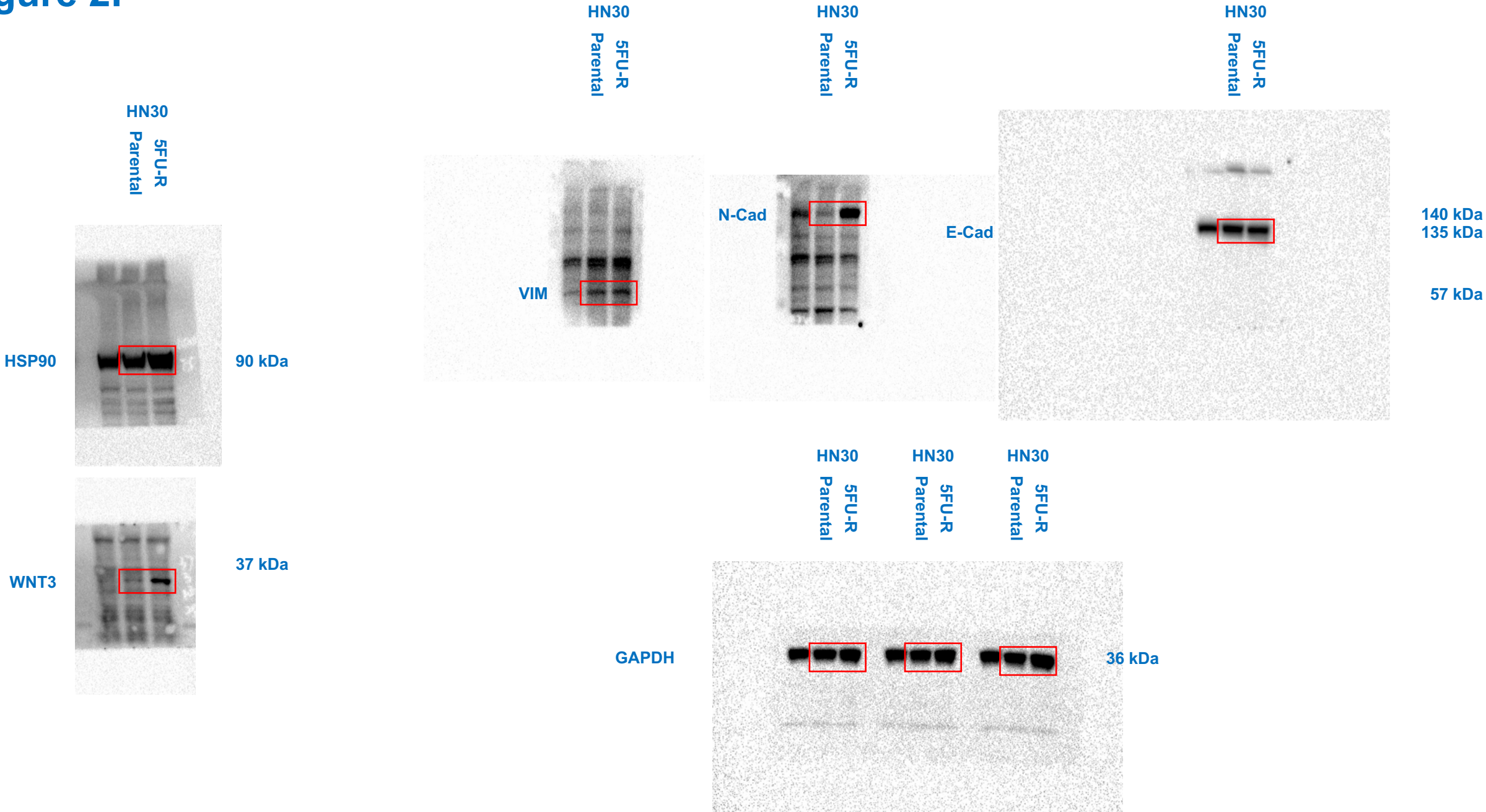

Figure 2H

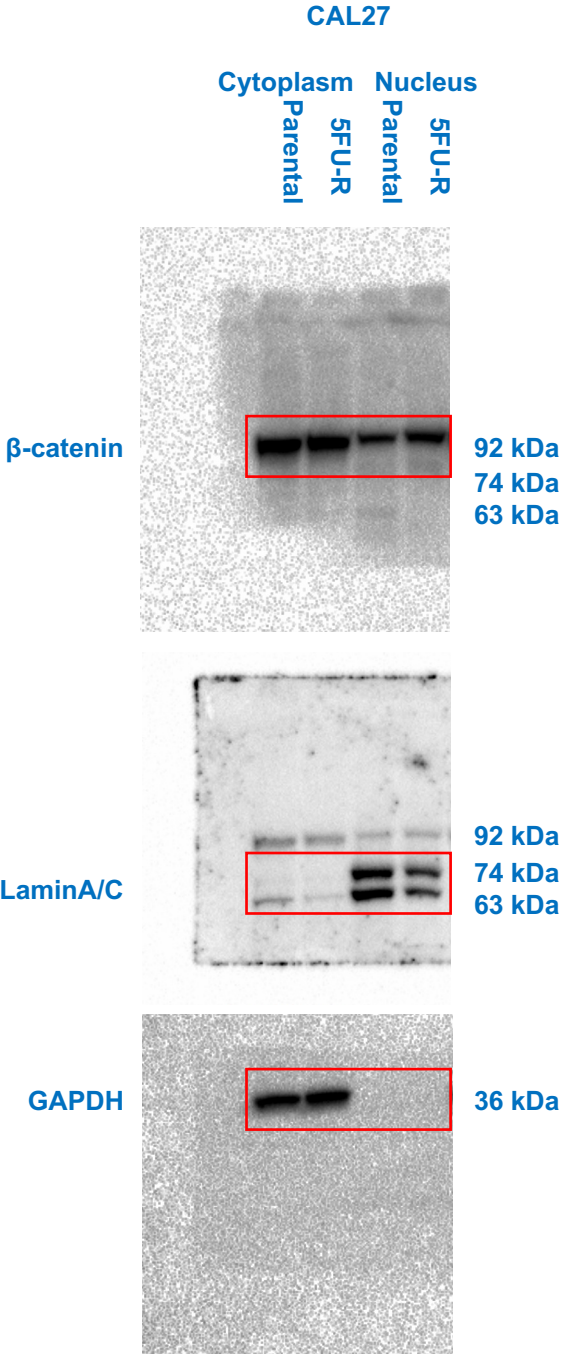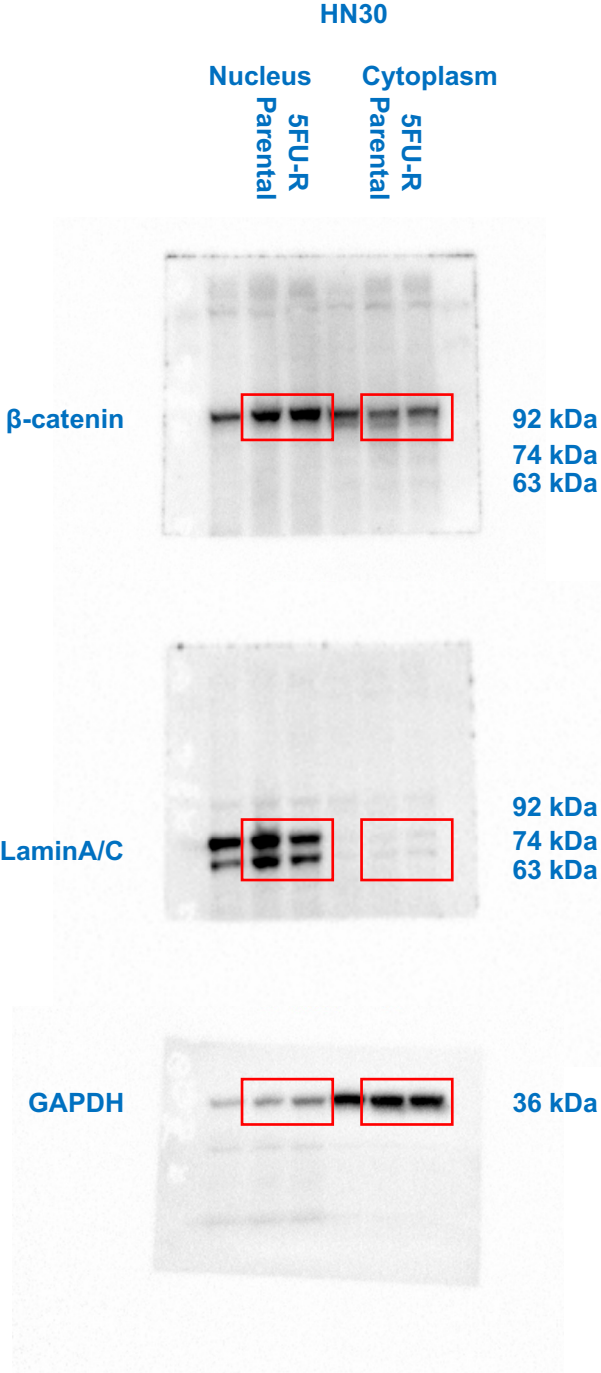

Figure 3B

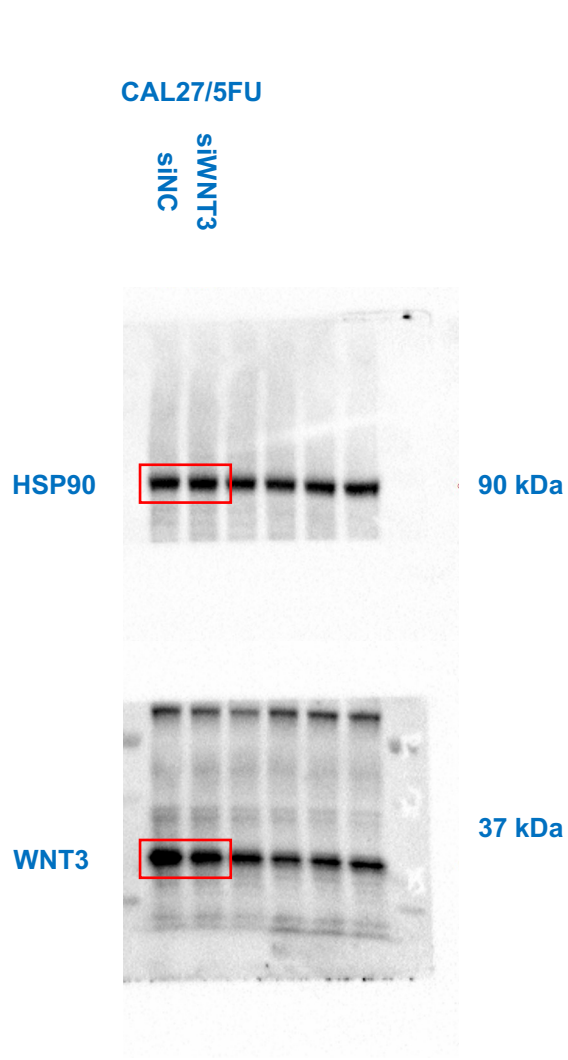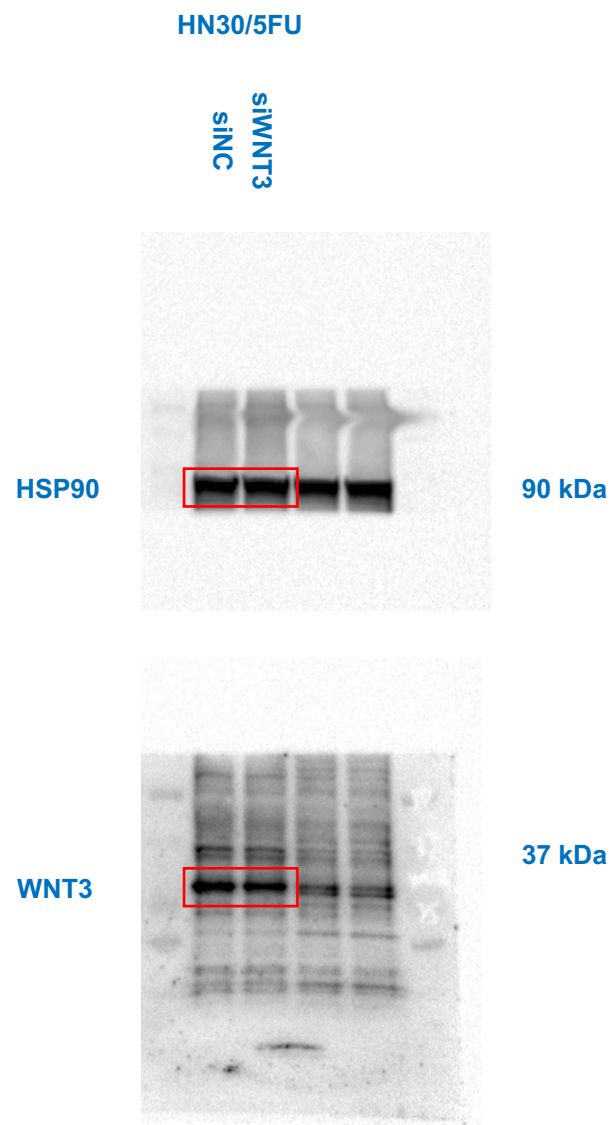

# Figure 4A

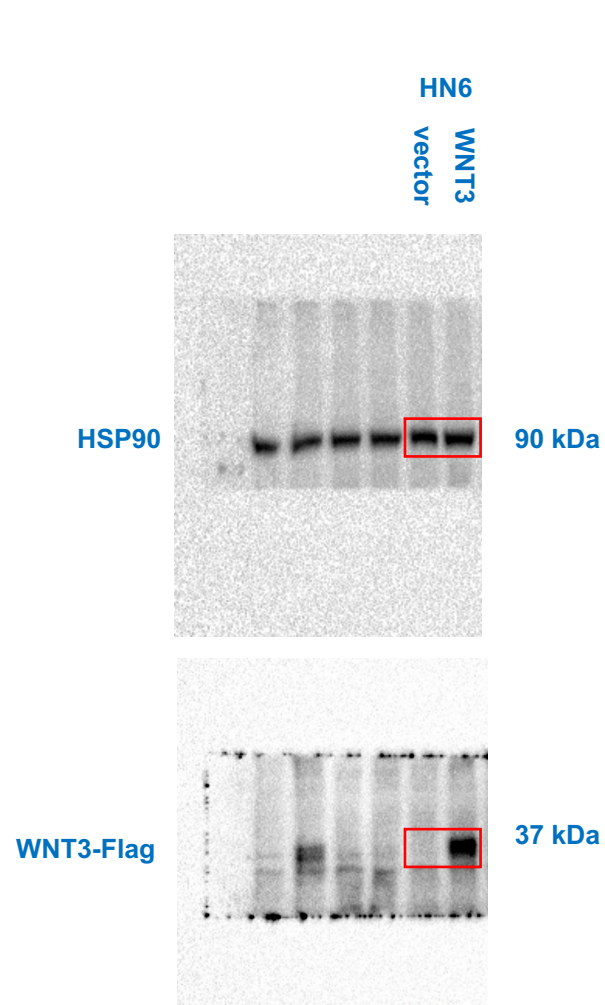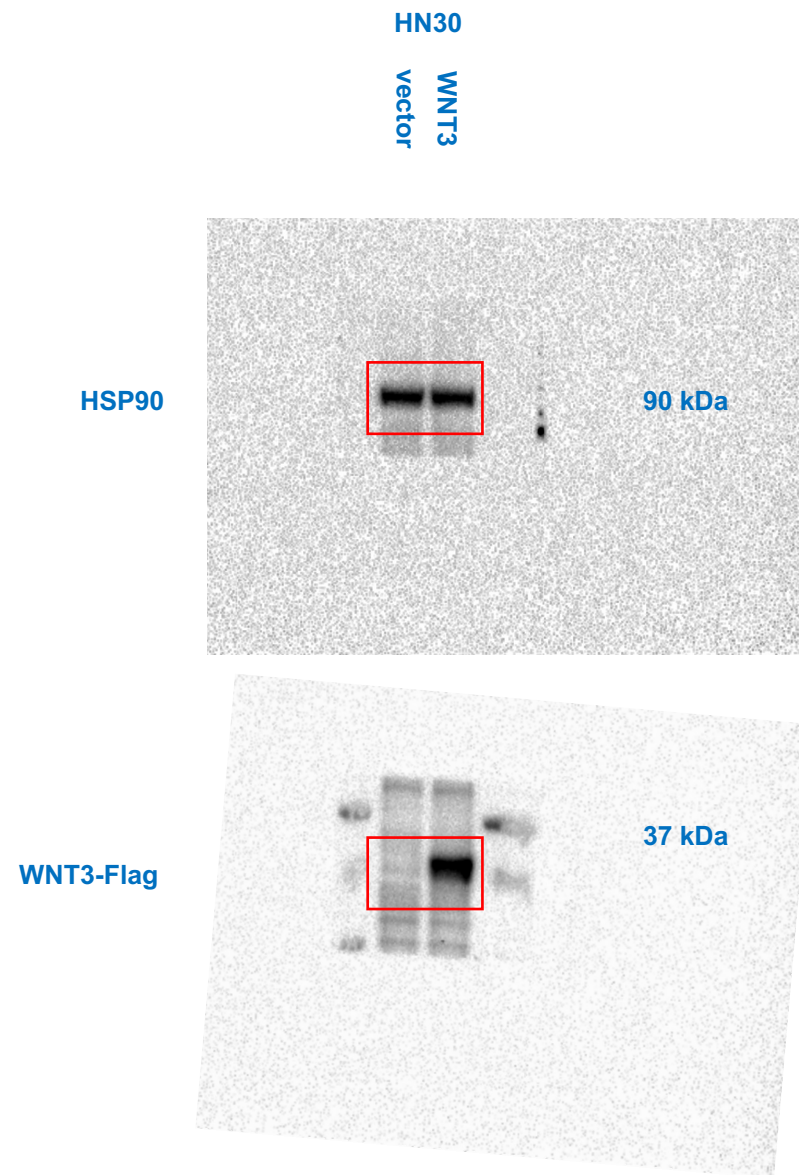

Figure 4J

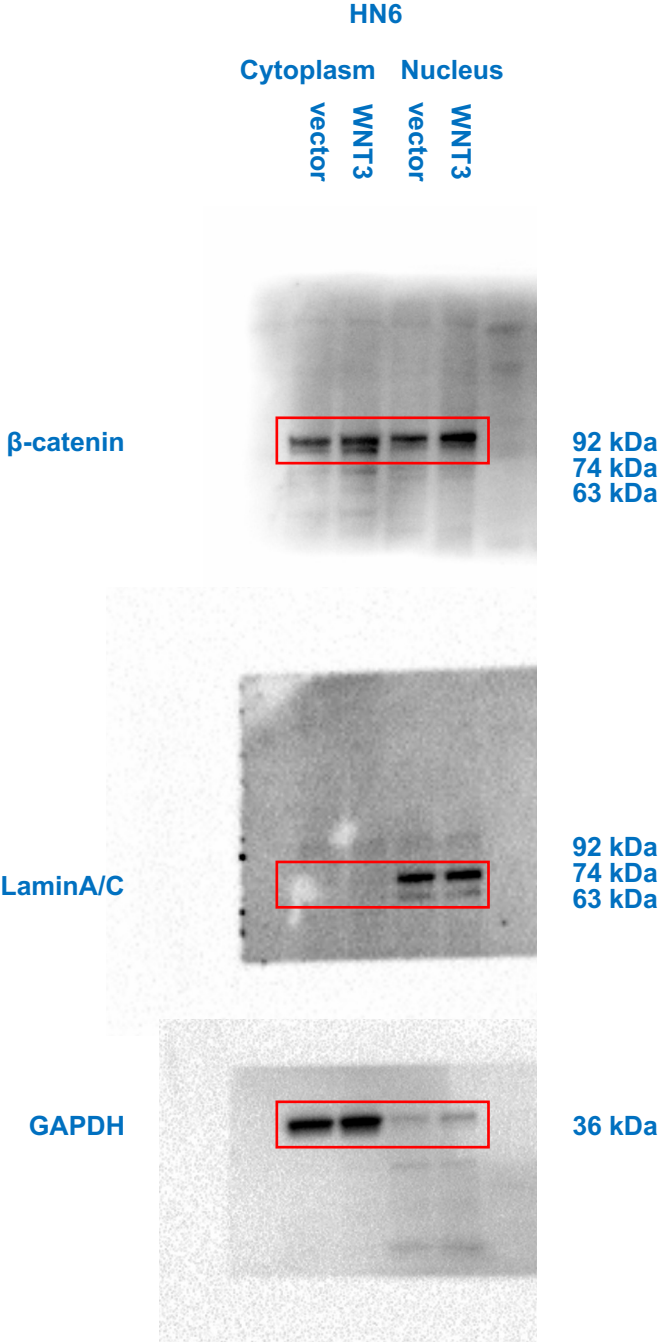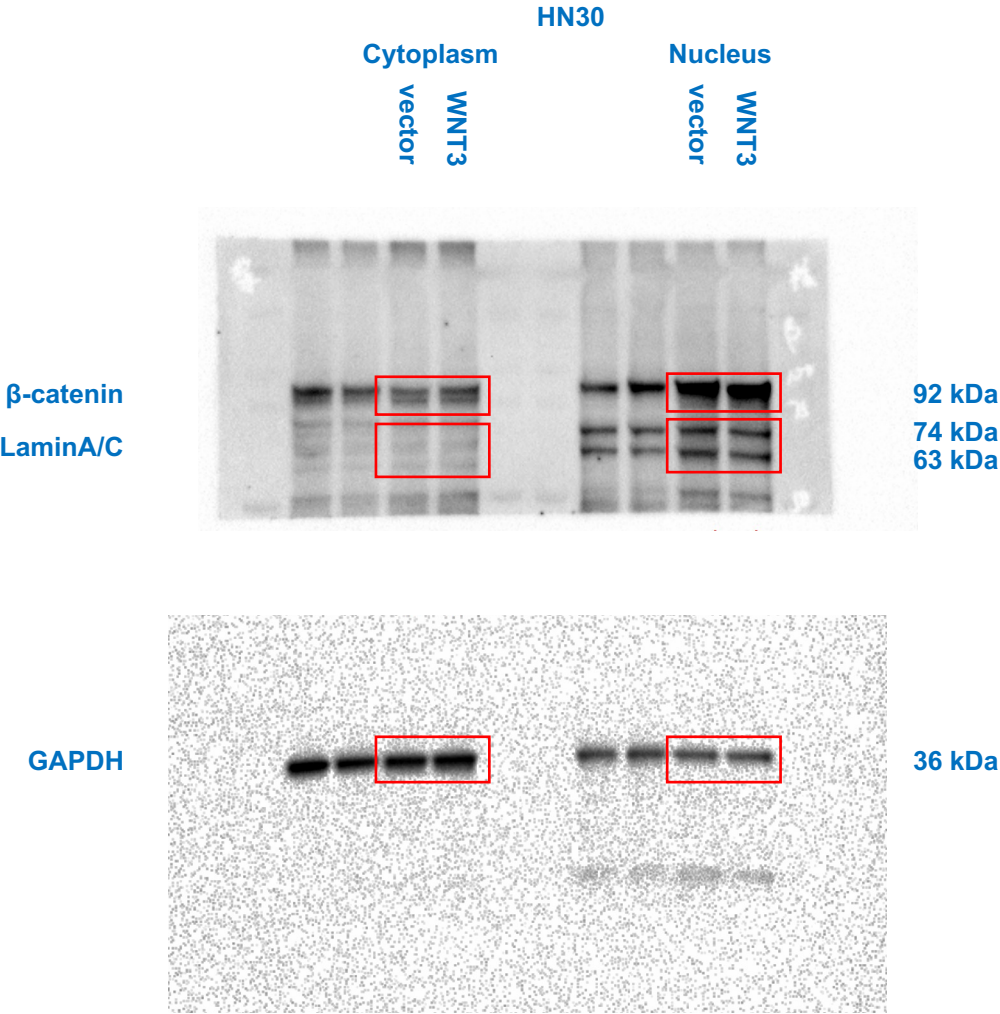

Figure 5C

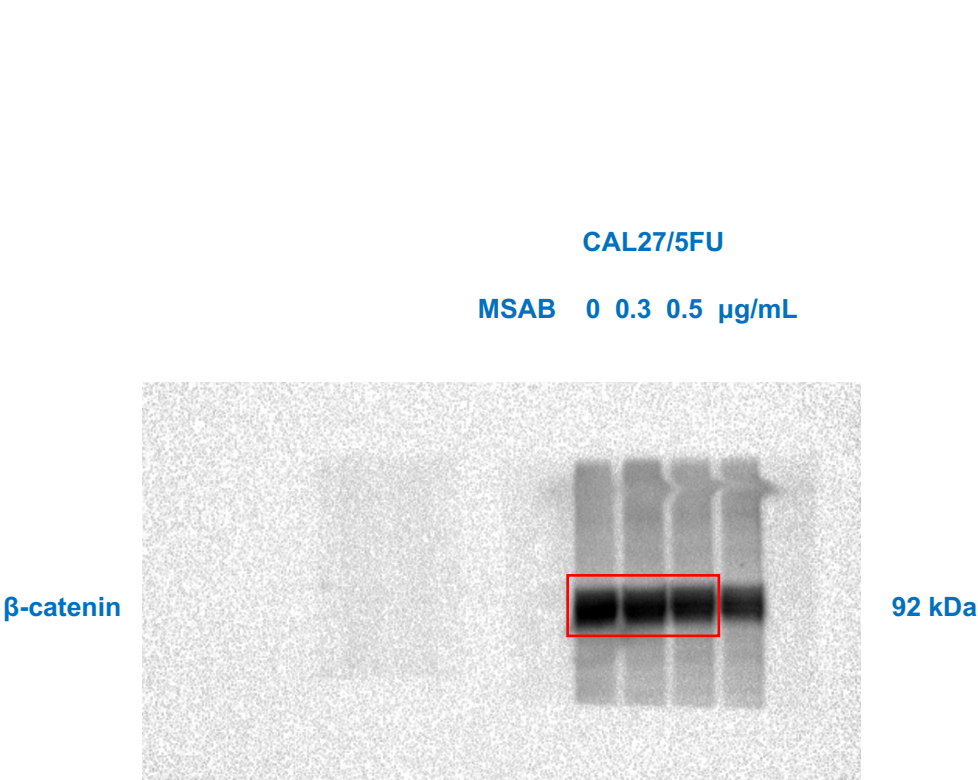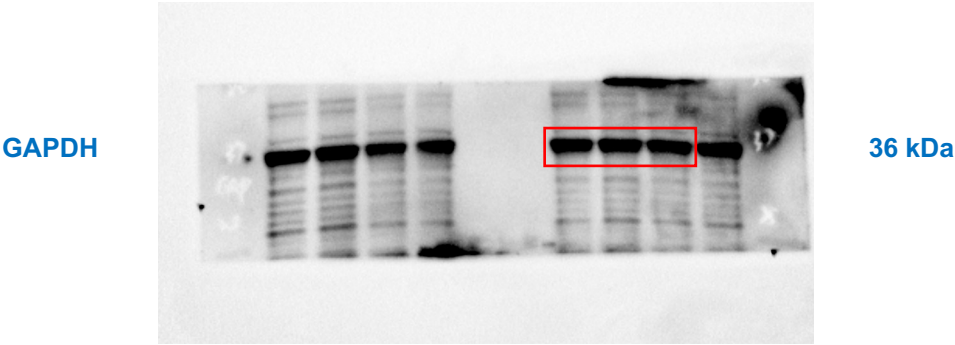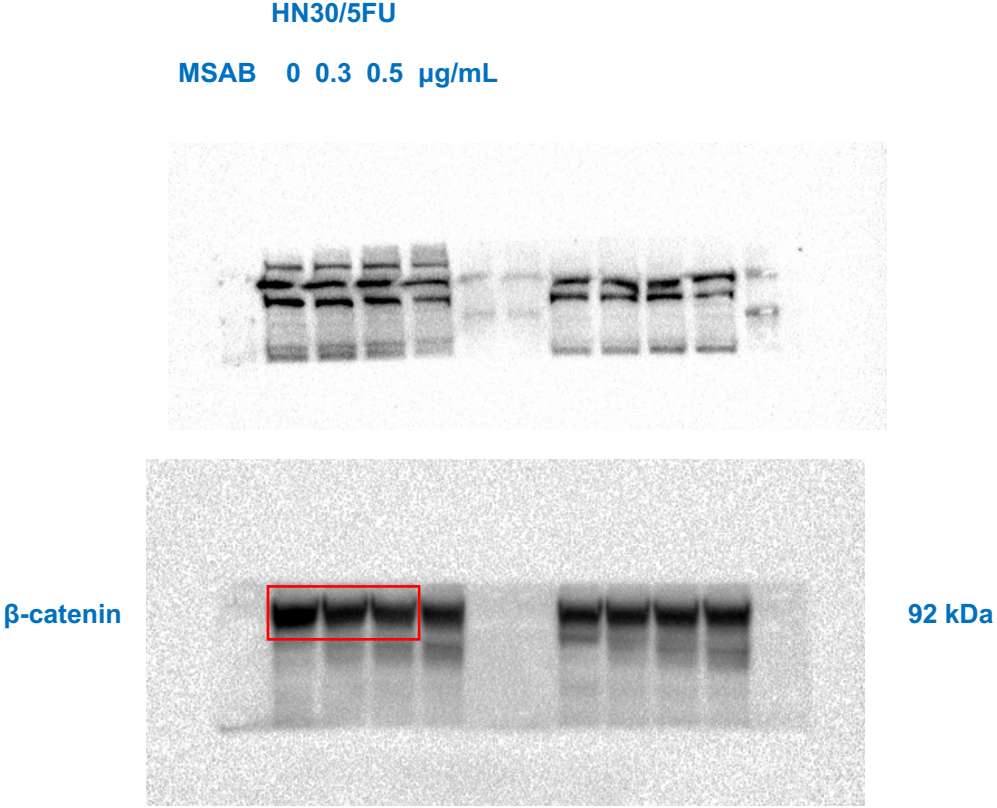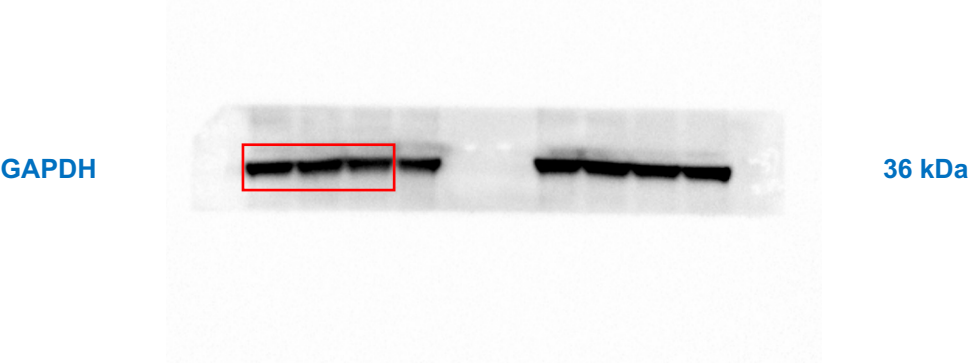

### Figure 5K

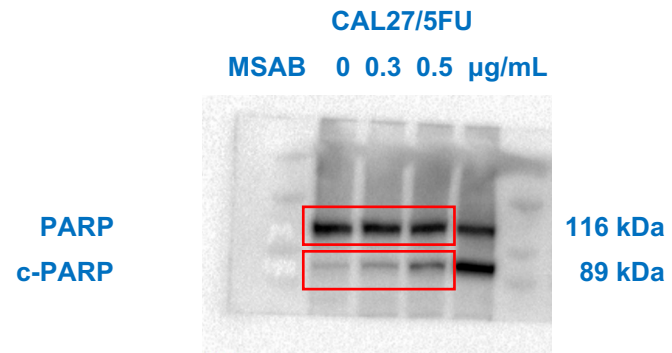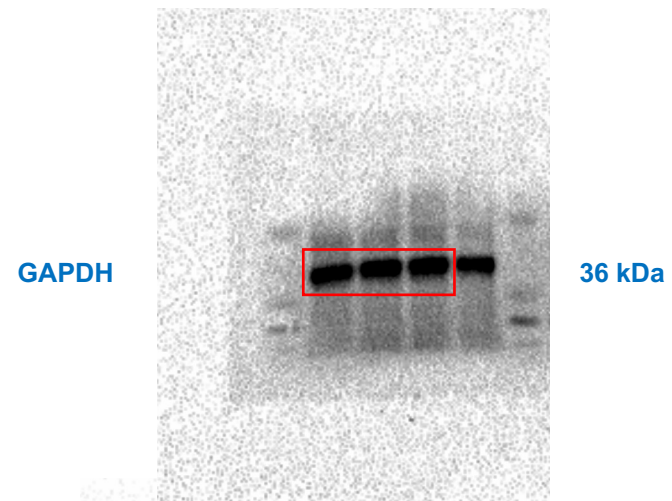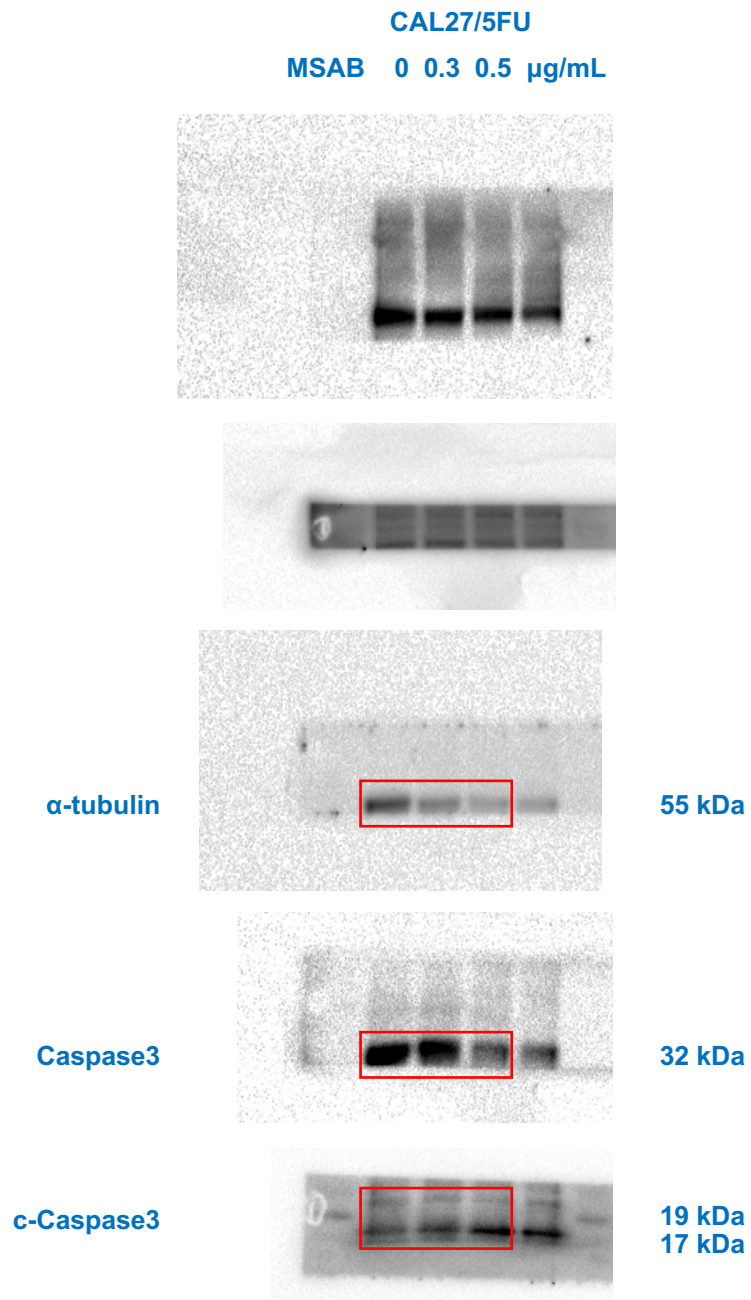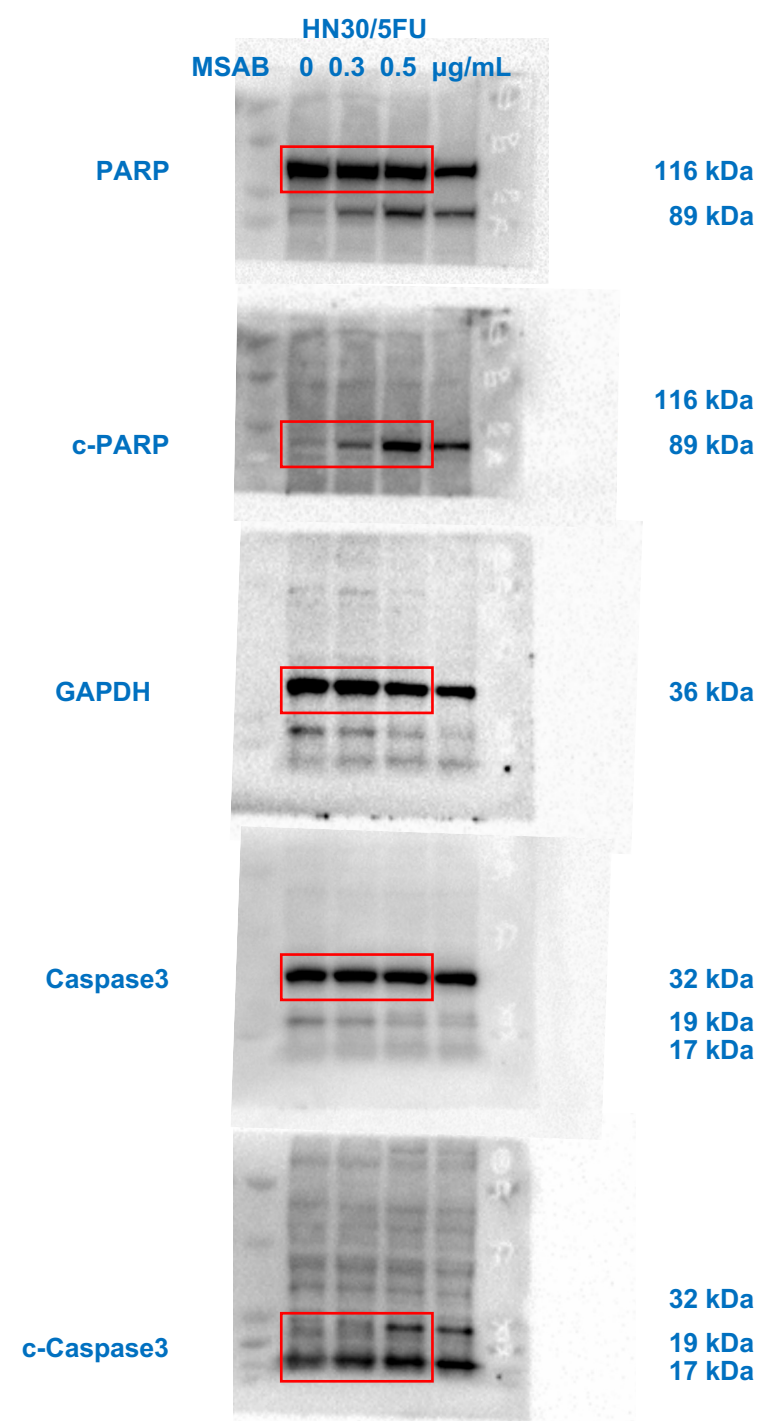

# Figure S2

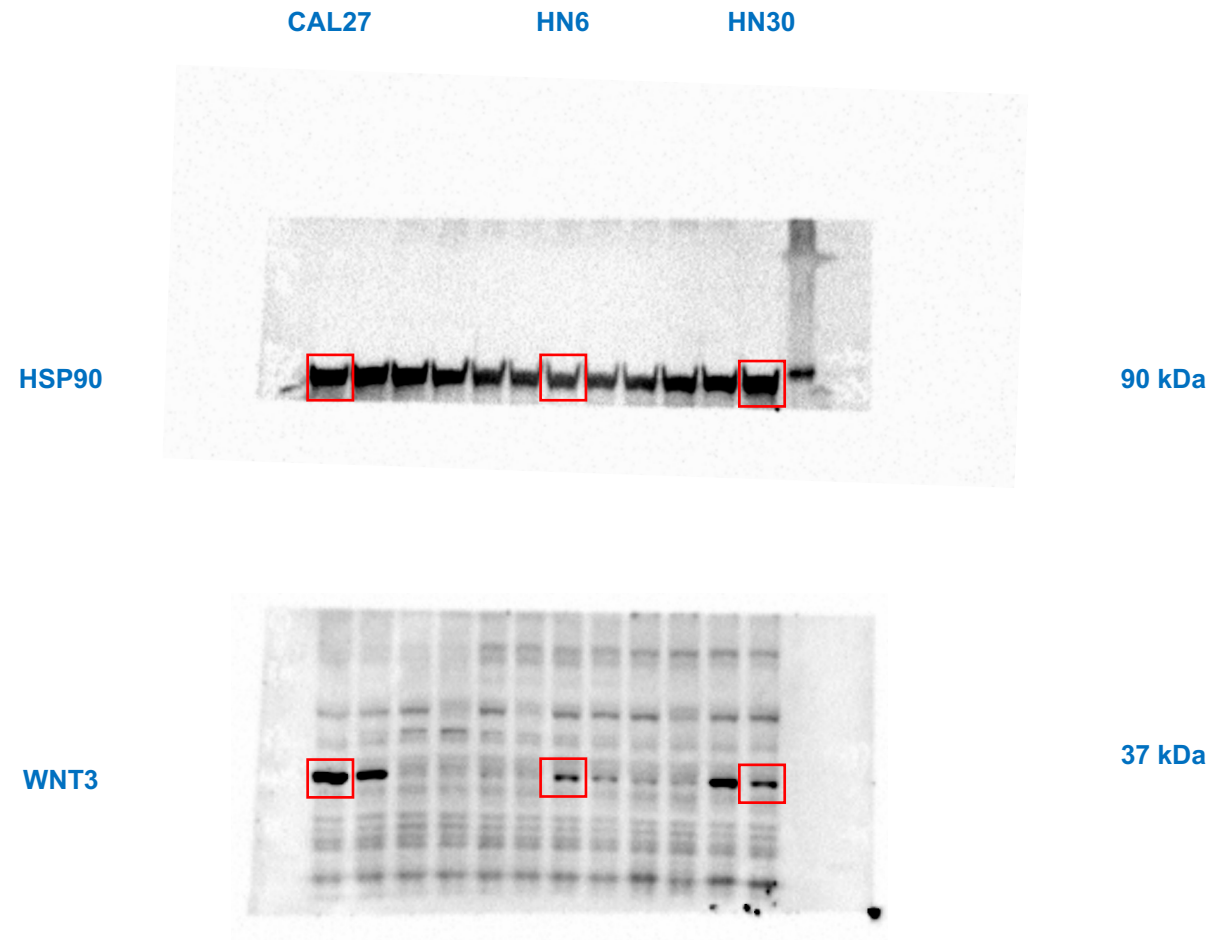

Supplement: Supplementary file 2 — Supplementary Material 2. [file 12885_2024_12318_MOESM2_ESM.pdf]
